# Ag(I) complexes with natural products and triphenylphosphine: activity against tumor cell lines and *mycobacterium tuberculosis*

**DOI:** 10.1007/s00775-026-02144-1

**Published:** 2026-04-28

**Authors:** Jocely Lucena Dutra, João Honorato de Araujo-Neto, Rafael Wendel Rodrigues Santana, Rone Aparecido De Grandis, Giulia Polinário, Fernando Rogério Pavan, Javier Ellena, Alzir Azevedo Batista

**Affiliations:** 1 Departament of Chemistry, Federal University of São Carlos (UFSCar), São Carlos, SP CEP 13561-905 Brazil; 2 Departament of Chemistry, Federal University of Amazonas (UFAM), Itacoatiara, AM CEP 69077-000 Brazil; 3 Department of Fundamental Chemistry, Institute of Chemistry, University of São Paulo (USP), São Paulo, SP CEP 05508-000 Brazil; 4 Department of Biological Sciences, School of Pharmaceutical Sciences of São Paulo State University (UNESP), Araraquara, SP CEP 14800-903 Brazil; 5 São Carlos Institute of Physics, University of São Paulo (USP), CP 369, São Carlos, SP CEP 13560-970 Brazil

**Keywords:** Silver(I) complexes, Natural products, Cytotoxicity, Anti-*mycobacterium tuberculosis*

## Abstract

Cancer and tuberculosis remain among the leading causes of morbidity and mortality worldwide, and the emergence of drug resistance in both diseases highlights the urgent need for new therapeutic strategies. In this context, metal-based compounds combined with bioactive natural products have emerged as promising alternatives to conventional drugs. In the present study, a series of silver (I) complexes containing natural products (curcumin, lawsone, lapachol or alizarin) and triphenylphosphine (PPh_3_) (complexes **1**–**4**) were synthesized and structurally characterized by IR spectroscopy, elemental analysis, molar conductivity, NMR (^1^H, ^13^C, ^31^P{^1^H}) spectroscopy and single crystal X-ray diffraction. The results of single crystal diffraction show that the two silver complexes are four-coordinated with two PPh_3_ and two O atoms of natural product, forming a distorted tetrahedral geometry. The in vitro cytotoxicity of silver(I) complexes was evaluated by MTT assay against four cancer cells and two normal cells, the result shows that the complexes presented a low IC_50_ value in general, including in normal cell lines. The complexes show better cytotoxic activity for A2780cis (cisplatin-resistant ovarian tumor cell line), being nearly 30 times higher than cisplatin. Silver complexes were tested against Mycobacterium tuberculosis (Mtb) reaching the threshold for effective antimicrobial activity. Furthermore, complex **4** showed good activity against clinical isolates of Mtb resistant to multiple first-line anti-TB drugs.

**Supplementary Information:**

The online version contains supplementary material available at 10.1007/s00775-026-02144-1.

## Introduction

Cancer is one of the most devastating diseases in the world, in these days, and is the second leading cause of death, killing more than 9 million people per year, according with world health organization (WHO). The diversity of cancer types and their resistance to treatment make the cure of the disease, a big challenging [1]. Although the chemotherapeutic drugs have achieved significant benefits and importance in cancer treatment, cellular resistance and several adverse effects, such as neurotoxicity, ototoxicity, and nephrotoxicity, are factors that limit the treatment of the disease. The discovery of cisplatin in 1960s was an unusual event in medicinal chemistry, mainly for cancer treatment [2]. Despite being effective against various cancers, the patient in treatment suffers from several side effects like nephrotoxicity, ototoxicity, and nausea, which occur because of lack of selectivity of the drug used in the cancer treatment [3]. The problem of toxicity and selectivity can be resolved by the help of the nanotechnology, but the problem of resistance acquired by cancer cells toward drugs requires a scientific solution, in the sense of the engineering of the compound that will be developed, to be used as drug [4, 5]. Thus, obtaining metal complexes with natural products, which present anticancer properties, as ligands, can be a good alternative for the generation of new drugs with improved antitumor activity. In this sense, silver-based complexes with natural products have emerged as good candidates to be explored as chemotherapeutic agents [6]. Thus, it is possible to modulate their properties for example by using phosphine ligands. It can also lead to better lipophilic properties of the complexes, facilitating their introduction of the drug into the cells [7]. 

Natural products have attracted the attention of the scientific community due to their recognized antitumoral activities. Natural products are generally widely distributed in nature and are commonly used to treat a variety of diseases [8, 9]. Some examples are naphthoquinones such as lawsone, (2-hydroxy-1,4 naphthoquinone) extracted from the leaves of *Lawsonia inermis* and lapachol (2-hydroxy-3-(3-methylbut-2-en-1-yl) naphthalene-1,4-dione) derived from plants of the Juglandaceae family [8]. Another example is the curcumin, (1,7-bis(4-hydroxy-3-methoxyphenil)1, 6-heptadiene-3,5-dione) extracted from the *Curcuma longa*, it is a compound present in mustard and curry, traditionally used in Indian cuisine [10]. Studies have shown that some of these compounds have been used to treat prostate cancer, lymphoma and leukemia [11]. In addition, anthraquinones such as alizarin (1,2-dihydroxyanthraquinone), when complexed with a metal, have demonstrated antitumor activity, in vitro, against a variety of tumor cell lines such as colon carcinoma, cervical breast cancer, hepatoma, and adenocarcinoma through a generation of reactive oxygen species (ROS)-mediated mechanism [8, 12]. 

The benefits of natural product complexation have been reported in several studies [8, 13, 14]. In fact, the pharmacological properties of natural products can be improved, by their coordination with a metal. Thus, it is possible to change the mechanism of a complex, modifying its interaction with DNA, modifying its lipophilicity, modifying its cellular target, etc. The engineering used in the construction of a complex can help to design metal-based drugs with enhanced anticancer activity and selectivity. Some studies have reported the complexation of lawsone and lapachol with a variety of metals such as Zn(II), Cu(II), Mn(II) and Ru(II), which exhibited good antitumor activity in human breast cancer cells [8]. In this sense, silver complexes need to be more explored for their potential as antimicrobial and antitumor agent [6, 15–17]. 

The coordination of a specific molecule to a metal allows to associate characteristics such as lipophilicity, redox propensity solubility in water, and in the biological medium. Studies have reported an improvement in the antitumor and antimicrobial activity of silver complexes in comparison with the free drug [16]. Various formulations of silver complexes have been tested against a wide range of diseases, and many of these formulations have included silver(I) complexes as antimicrobial and anticancer agents [15–17]. A good example in this scenario is Tuberculosis (TB), one of the deadliest diseases caused by a single infectious agent in the world. Despite the efficacy of medical drugs treatment against TB, the bacterial resistance and the time of treatment, continues to be a problem. This way, a good alternative to counteract this problem is to develop drugs capable of rapidly eliminating the bacterial population and avoiding the appearance of multidrug resistance cases such as MDR-TB and XDR-TB [17]. 

Here we describe the synthesis of four silver(I) complexes containing lawsone, lapachol, curcumin and alizarin as ligands, and the biological studies against tumor and non-tumor cell lines. The triphenylphosphine was also used as ligand, to complete the coordination sphere of these complexes, with the intention to improve their lipophilicity. In addition, the compounds were preliminarily tested against tuberculosis.

## Materials and methods

### General remarks

All the syntheses were performed under an argon atmosphere (White Martins^®^), to avoid the presence of oxygen and moisture. All chemicals used to prepare the complexes and buffer solutions were of analytical grade or chemically pure grade. The AgClO_4_ and triphenylphosphine (PPh_3_) were used as received from Aldrich. Elemental analyses (CHN) were performed at the Analytical Laboratory at the Federal University of São Carlos (Brazil/SP) using an EA 1108 CHNS microanalyzer (Fisons Instruments^®^). FTIR spectra were obtained using a Bomem- Michelson^®^ 102 spectrometer in the 4000–400 cm^− 1^ region with KBr sample pellets. Conductivity measurements in DMSO solution (1.0 mM) of the complexes were performed at 298 K, using a Meter Lab^®^ model CDM230 conductivity meter. Optical spectra were recorded on an HP8452A (diode array) spectrophotometer, in DMSO solutions (UV Cutoff of 190 nm) with a 1.0 cm quartz cell in the range of 200–800 nm. NMR experiments were carried out using 9.4 T BRUKER^®^ equipment (400 MHz for hydrogen frequency), in acetone-d_6_. ^31^P{^1^H} NMR spectra were recorded in CH_2_Cl_2_ using a capillary containing D_2_O. All chemicals shifts (*δ*) are given in ppm.

### Synthesis

#### Precursor synthesis

The precursor complex [Ag(PPh_3_)_2_(CH_3_CN)]∙ClO_4_ was synthesized following the method previously described, with adaptions [18]. In a dark room and argon atmosphere, AgClO_4_ (0.96 mmol; 0.200 g) was dissolved in 15 mL of acetonitrile. A solution of triphenylphosphine (1.92 mmol; 0.506 g) in 40 mL of acetonitrile was added to this. After stirring manually to ensure complete mixing of the solution, it was left to rest for 90 min. The reaction was evaporated to dryness then 25 mL of CH_2_Cl_2_ was added and then hexane was added little by little until a white solid precipitated. The solid was filtered and dried under *vacuum*. ^31^P{^1^H} NMR (CH_2_Cl_2_(D_2_O)): 12.7 ppm (d, *J* = 516.7 Hz). Yield: 82%.

#### Syntheses of the complexes with the general formula [Ag(PPh_3_)_2_(O-O)

In an argon atmosphere 0.16 mmol of the ligand (for **1**, curcumin (cur), 24.0 mg; for **2**, lawsone (law) 13.5 mg; for **3**, lapachol (lap) 15.1 mg; for **4**, alizarin (ali) 15.5 mg) was dissolved in 5 mL of methanol and Et_3_N (15 µL) was added. After 5 min of stirring, the precursor complex [Ag(PPh_3_)_2_CH_3_CN]ClO_4_ (0.15 mmol; 50.0 mg) was added. After 4 h of stirring, the volume was reduced for 2 mL and water was added to obtained a solid, which was filtered off, washed with hot hexane and dried under *vacuum*.

[Ag(PPh_3_)_2_(cur)] (**1**) Orange solid; yield: 42% (24.0 mg); Anal. calc. for C_57_H_49_AgO_6_P_2_: C, 68.47; H, 4.94. Found: C, 68.09; H, 5.08%. Molar conductance (S cm^2^ mol^-1^, DMSO): 16.5. IR (KBr, cm^-1^): 3450 ν(O-H); 1620 ν(C = O); 1282 ν(C-O);1122 ν(P-CPh). ^1^H NMR (600 MHz, CD_3_CN, 298 K): δ 7.56 (t; 2H; *J* = 16.5 Hz; cur_(4)_); 7.42 (dt; 4 H; *J* = 7.4, 1.0 Hz; PPh_3_); 7.26 (d; 2H; *J* = 1.8 Hz; Ph cur_(6)_); 7.23 (dd; 12 H; *J* = 7.9, 1.6 Hz; PPh_3_); 7.15 (s; 2H; Ph cur_(10)_); 7.15–7.09 (m; 12 H; PPh_3_); 6.86 (d; 4 H; *J* = 8.2 Hz; Ph cur_(9)_); 6.68 (d; 2H; *J* = 15.8 Hz; cur_(3)_); 5.92 (s; 1H; cur_(1)_); 3.91 (s; 6 H; cur_(11)_). ^13^C NMR (151 MHz, CD_3_CN) δ 184.59 (2), 149.55 (8), 148.57 (7), 141.28 (4), 134.41 (PPh_3_), 132.87 (PPh_3_), 131.46 (PPh_3_), 129.99 (PPh_3_), 128.42 (5), 123.98 (10), 122.56 (3), 115.98 (6), 111.49 (9), 102.04 (1), 56.68 (11). ^31^P{^1^H} NMR (CH_2_Cl_2_ (D_2_O)): 10.2 ppm (s).

[Ag(PPh_3_)_2_(lau)] (**2**) Orange solid; yield: 75% (39.0 mg); Anal. calc. for C_46_H_35_AgO_3_P_2_: C, 68.58; H, 4.38. Found: C, 68.62; H, 4.58%. Molar conductance (S cm^2^ mol^-1^, DMSO): 15.7. IR (KBr, cm^-1^): 1676 ν(C = O); 1278 ν(C-O); 1093 ν(P-CPh). ^1^H NMR (400 MHz, CDCl_3_, 298 K): δ 8.06 (dd; 1H; *J* = 7.7, 0.8 Hz; H_law(8)_); 7.76 (dd; 1H; *J* = 7.6, 0.9 Hz; H_law(5)_); 7.61 (td; 1H; *J* = 7.5, 1.3 Hz; H_law(7)_); 7.44 (td; 1H; *J* = 7.5, 1.3 Hz; H_law(8)_); 7.41–7.24 (m, 34 H; PPh_3_). ^13^C{^1^H} NMR (100 MHz, CDCl_3_, 298 K): δ 188.03 (1), 184.44 (4), 170.27 (2), 135.36 (7), 133.81 (PPh_3_), 131.89 (6), 130.51 (PPh_3_), 129.10 (PPh_3_), 125.72 (8,5), 109.37 (3). ^31^P{^1^H} NMR (CH_2_Cl_2_ (D_2_O)): 9.8 ppm (s).

[Ag(PPh_3_)_2_(lap)] (**3**) Purple solid; yield: 75% (43 mg); Anal. calc. for C_51_H_43_AgO_6_P_2_: C, 70.11; H, 4.96. Found: C, 69.98; H, 5.12%. Molar conductance (S cm^2^ mol^-1^, DMSO): 12.1. IR (KBr, cm^-1^): 3465 ν(O-H); 1645 ν(C = O); 1274 ν(C-O); 1091 ν(P-CPh). ^1^H NMR (400 MHz, CDCl_3_, 298 K): δ 8.02 (d; 1H; *J* = 7.6 Hz; H_lap(8)_); 7.64 (d; 1H; *J* = 7.5 Hz; H_lap(5)_); 7.51 (t; 1H; *J* = 7.5 Hz; H_lap(7)_); 7.46–7.21 (m; 31H; H_lap(6)_ and PPh_3_); 5.34 (t, 1H; *J* = 6.6 Hz; H_lap(12)_); 3.38 (d, 2H; *J* = 6.7 Hz; H_lap(11)_); 1.70 (d, 6 H; *J* = 72.5 Hz; H_lap(14,15)_). ^13^C{^1^H} NMR (100 MHz, CDCl_3_, 298 K): δ 187.92 (1), 182.41 (4), 168.03 (2), 135.57 (6), 133.99 (PPh_3_), 133.50, 132.24 (PPh_3_), 130.19 (PPh_3_), 129.84, 128.95 (PPh_3_), 125.65, 125.12, 124.31, 121.34 (12), 25.98 (14), 23.18 (11), 18.20 (15). ^31^P{^1^H} NMR (CH_2_Cl_2_ (D_2_O)): 9.2 ppm (s).

[Ag(PPh_3_)_2_(ali)] – (**4**) Purple solid; yield: 80% (45 mg); Anal. calc. for C_50_H_37_AgO_4_P_2_: C, 68.90; H, 4.28. Found: C, 68.93; H, 4.36%. Molar conductance (S cm^2^ mol^-1^, DMSO): 15.3. IR (KBr, cm^-1^): 1625 ν(C = O); 1255 ν(C-O); 1089 ν(P-CPh). ^1^H NMR (400 MHz, CDCl_3_, 298 K): δ 8.28 (d; 1H; *J* = 7.4 Hz; H_ali(11)_); 8.20 (d, 1H, *J* = 7.4 Hz, H_ali(8)_); 7.73 (t, 1H, *J* = 7.4 Hz, H_ali(4)_); 7.71–7.64 (m, 2H, H_ali(9,10)_); 7.38 (t, 8 H, *J* = 6.8 Hz, PPh_3_); 7.33–7.20 (m, 31H, PPh_3_); 6.96 (d, 1H, *J* = 8.4 Hz, H_ali(3)_). ^13^C NMR (101 MHz, CDCl_3_, 298 K): δ 189.71 (13), 180.53 (1), 163.08 (6), 153.88 (2), 135.39, 134.15, 133.84 (PPh_3_), 132.57, 131.25, 130.72 (PPh_3_), 129.18 (PPh_3_), 127.12, 126.40, 123.85, 121.35, 119.52, 115.80. ^31^P{^1^H} NMR (CH_2_Cl_2_ (D_2_O)): 10.6 ppm (s).

### Single-crystal X-ray diffraction

The complexes **2** and **3** were crystallized from methanol solutions through the slow evaporation of the solvent. Single-crystal X-ray diffraction data were collected using a Rigaku XtaLAB mini diffractometer equipped with graphite monochromated Mo Kα radiation (λ = 0.71073 Å). Unit cell parameters were refined using the CrysAlisPro software suite. Structure determination was carried out via intrinsic phasing, utilizing the SHELXT program [19]. Absorption corrections were applied using the Gaussian method. Structural illustrations and tabulated data were prepared using OLEX2 [20] and MERCURY [21], respectively. Crystallographic data for the analyzed structures have been deposited in the Cambridge Crystallographic Data Centre (CCDC). These data can be accessed at https://www.ccdc.cam.ac.uk/structures by searching for the specific CCDC deposition codes (complex **2–2415261**, complex **3–2415260**). The corresponding checkCIF reports (checkcif-2415260.pdf and checkcif-2415261.pdf) and the mercury files (2415261 and 2415260) are provided as Supplementary Materials.

### Solution stability evaluation

Since the experiments are performed with previous solubilization of the complexes in DMSO, the stability of the complexes **1**–**4** was evaluated in this solvent, using ^31^P{^1^H} NMR spectroscopy. The ^31^P{^1^H} NMR spectra of the complexes in dimethyl sulfoxide (with a deuterated water capillary) were recorded at 0, 24, and 48 h.

### Cell viability assay in vitro

#### Cell culture

The cell lines employed in these assays were the human breast tumor cell lines MDA-MB-231 (ATCC No. HTB-26) and MCF-7 (ATCC No. HTB-22), lung tumor cell line A549 (ATCC No. CCl-185), cisplatin-resistant ovarian tumor cell line A2780cis (Sigma 93112517) and the non-tumor human breast and lung cell lines MCF-10 A (ATCC No. CRL-10317) and MRC-5 (ATCC No. CCL-171), respectively. The cells were routinely maintained at 37 °C in a humidified 5% CO_2_ in Dulbecco’s Modified Eagle (DMEM) medium containing fetal bovine serum (FBS) 10% (MDA- MB-231, A549 and MRC-5), Roswell Park Memorial Institute 1640 medium (RPMI 1640) containing FBS 10% (MCF-7 and A2780cis) and DMEM/F12 medium containing horse serum (HS) 5%, epidermal growth factor (EGF − 0.02 mg mL^˗1^); hydrocortisone (0.05 mg mL^˗1^); cholera toxin 0.001 mg mL^˗1^) and insulin (0.01 mg mL^˗1^) (MCF-10 A). All media contained penicillin (100 UI mL^˗1^), streptomycin (100 mg mL^˗1^) and L-glutamine (2 mM).

#### MTT assay

For the cell viability assay, 1.5 × 10^4^ cells/well were seeded in 150 µL of complete medium in 96-well plates (Corning Costar) and incubated at 37 °C under 5% CO_2_ for 24 h to allow adhesion. After that, the compounds were added at different concentrations (0.04, 0.15, 0.62, 2.50, 10.00, 20.00, 30.00 and 40.00 µmol L^− 1^), dissolved in DMSO (0.5%). The cells were incubated with the compound at 37 °C under 5% CO_2_ for 48 h. After that, the MTT [3-(4,5-dimethylthiozol-2-yl)-2,5-diphenyltetrazolium bromide] solution (1 mg mL^− 1^, 50 mL per well) was added to the cells, and the cells were incubated for 4 h. Next, 150 mL of isopropanol was added to dissolve the precipitated formazan crystals. MTT conversion to formazan by metabolically viable cells was measured on an automated microplate reader at 540 nm. The viability rate in the control wells (cells receiving only DMSO) was taken as a reference (100%) of cell viability, and the viability rates of the treated cultures were expressed as a percentage of the control value. The percentage of cell viability was plotted against drug concentration (logarithmic scale) to determine IC_50_ (the drug concentration at which 50% of the cells are viable relative to the control). The error was estimated from the average of 3 trials, by Hill’s equation in the GraphPad Prism 9.0 software.

#### Anti- *Mycobacterium tuberculosis* activity

The anti-*MTB* activity of the compounds was determined by the REMA (Resazurin Microtiter Assay) method [22]. Assays were performed in 96-well plates (lines A-H, columns 1–12) and each well was filled with 100 µL of 7H9 medium (Sigma-Aldrich) supplemented with 10% OADC, followed by 100 µL of each of the compounds diluted in the wells of column B. After homogenization, an aliquot of 100 µL of the mixture was removed and transferred to the next well, performing serial dilutions in the 1:1 ratio successively from column 2 to column 10, and discarding the final 100 µL. A volume of 100 µL of the diluted bacterial inoculum was added to each well making up a final volume of 200 µL, the final concentration of substance tested in column 2 was 25 µg/ml and in column 10 it was 0.09 µg/ml. In column 1, a sterility control of each tested compound was performed; column 11 was the positive control of the bacteria inoculum, and column 12 was the sterility control of the culture medium.

The bacteria dilution was made from the culture with turbidity of 1.0 McFarland, diluted in 1:100, through dilution calculation, considering 1.0 McFarland = 3 × 10^8^ UFC/ml. As a positive control, a rifampicin solution at an initial concentration of 1 µg/mL was used, as rifampicin has a MIC_90_ around 0.04 µg/mL. The microplates were incubated in a humidified greenhouse, 37 °C, 5% CO_2_ for 7 days. After this period, resazurin developer was applied at a concentration of 0.01%, and only after 24 h was performed the fluorescence reading with excitation at 530 nm and emission at 590 nm in the Cytation 3 (Biotek) equipment. Resazurin is a revealer that allows the observation of cell viability through colorimetric change (qualitative) and fluorescence emission (quantitative). The MIC was defined as the lowest concentration resulting in 90% inhibition of *M. tuberculosis* growth.

#### Selectivity index

In cytotoxicity assays, the selectivity index (SI) was calculated by dividing the IC_50_ normal cells/IC_50_ tumoral cells, which demonstrates how much a compound is selective in killing the cancer cells without causing damage in normal cells.

**Fig. 1:**
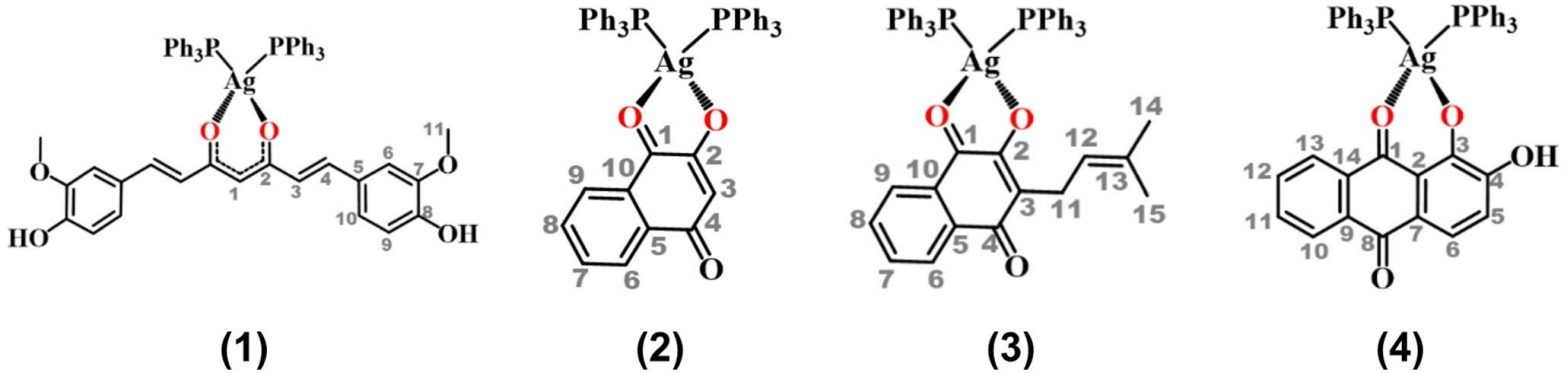
Numbering scheme of complexes **1–4** for NMR data

In Mtb assays, the SI was calculated by dividing the IC_50_(MRC-5) /MIC_90_ values, which demonstrates how much a compound is selective in killing the microorganism without causing cellular damage, in which a promising value is SI *>* 10 [23]. Fig. 1.

## Results and discussion

### Characterization

**Scheme 1:**
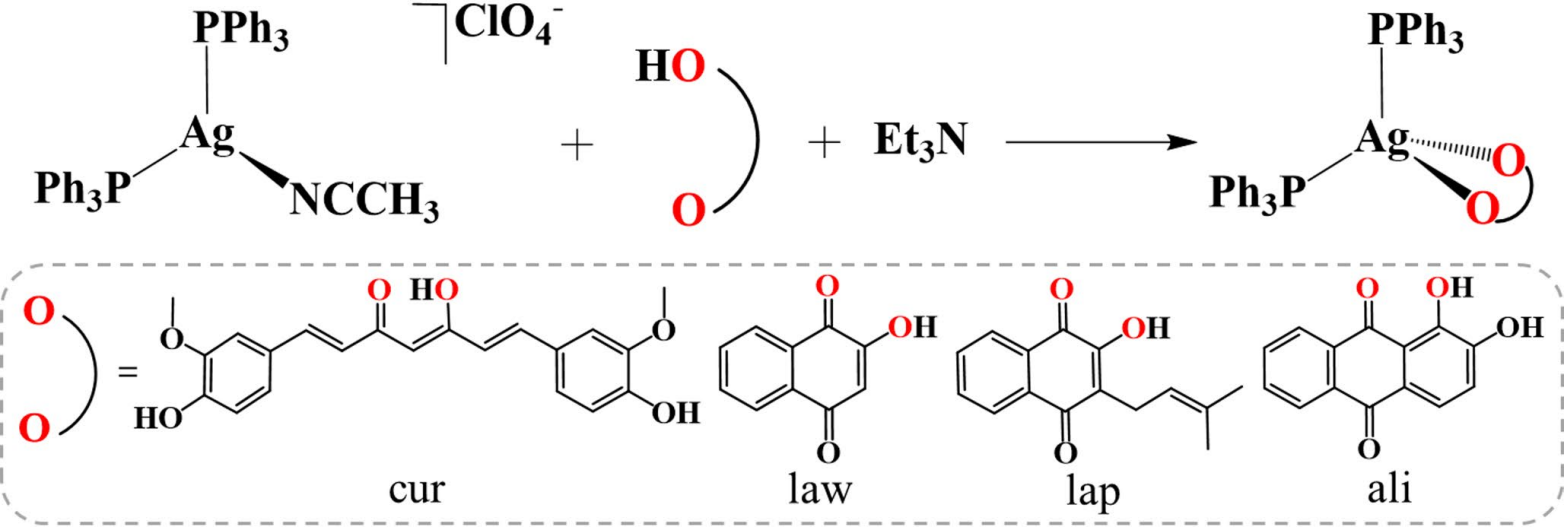
Representative scheme of the reaction path of the Ag(I) complexes with natural products ligands

The complexes **1–4** were synthesized from the reaction of the precursor [Ag(PPh_3_)_2_(CH_3_CN)]∙ClO_4_, synthesized previously, and the ligand [curcumin (cur); lawsone (law); lapachol (lap) or alizarin (ali)] deprotonated with Et_3_N in a 1:1 molar ratio, respectively (Scheme 1). The compounds were characterized by IR and NMR spectroscopy, molar conductance, elemental analysis and X-ray diffraction.

Microanalysis data confirmed the expected formulation. The molar conductance measurements were carried out in DMSO and showed values below the range expected for 1:1 electrolytes (21.6–26.8 S cm² mol⁻¹), with values of 16.5, 15.7, 12.1, and 15.3 S cm² mol⁻¹ for complexes 1–4, respectively, indicating that the complexes are neutral. The IR spectra of **1–4** showed characteristics bands at approximately 1590 cm^− 1^ *v*(C = O) has moved to lower frequency that in the ligands, this indicated coordination of carbonyl group with the silver (Table 1) [24, 25]. The presence of intense vibrational band at 1095 cm^− 1^ evidenced coordination of triphenylphosphine in the compounds **1–4** (Fig. S1) [16, 26]. Infrared spectra corresponding to the ligands are displayed in Fig. S2–S5.

The ^31^P{^1^H} NMR chemical shift changes from the precursor (12.7 ppm) (Fig. S6, and Fig S7 for triphenylphosphine) to the synthetized complexes, by approximately 10 ppm, is evidence of the change in the coordination sphere of the Ag(I) center (Fig. S8). The ^31^P{^1^H} NMR chemical shifts are listed in Table 1.

We characterized all the complexes by 1D/2D ^1^H and ^13^C NMR spectroscopies (Fig. S9-S13). The ^13^C NMR spectra of all complexes (Fig. S10) showed the signals related to C = O and C$\:-$O carbon atoms appeared in downfield upon coordination in comparison with the free ligands. The shift of these signals to a low field is a consequence of the coordination of the molecule with the Ag(I). In complex **1** there is not a large displacement of this signal due to the resonance presented in the six-membered ring formed after coordination (Table 1).

**Table 1:** NMR chemical shift data and main bands (cm^− 1^) in the infrared spectra (KBr pellets) for the ligands and complexes with the general formula [Ag(PPh_3_)_2_(O-O)]

	NMR (ppm)			FTIR (cm^− 1^)
	^31^*P*{^1^H}	^13^C (C = O)	^13^C (C$\:-$O)	ν (C = O)
**1**	10.2 (s)	184.6	-	1620
Cur	-	184.5	-	1629
**2**	9.8 (s)	188.0	170.3	1676
Law	-	182.1	156.5	1679
**3**	9.2 (s)	187.9	168.0	1645
Lap	-	181.8	152.8	1658
**4**	10.6 (s)	189.7	163.1	1625
Ali	-	182.1	151.0	1664
Prec	12.7 (d)	-	-	-

Prec = [Ag(PPh_3_)_2_CH_3_CN]ClO_4_

**Fig. 2:**
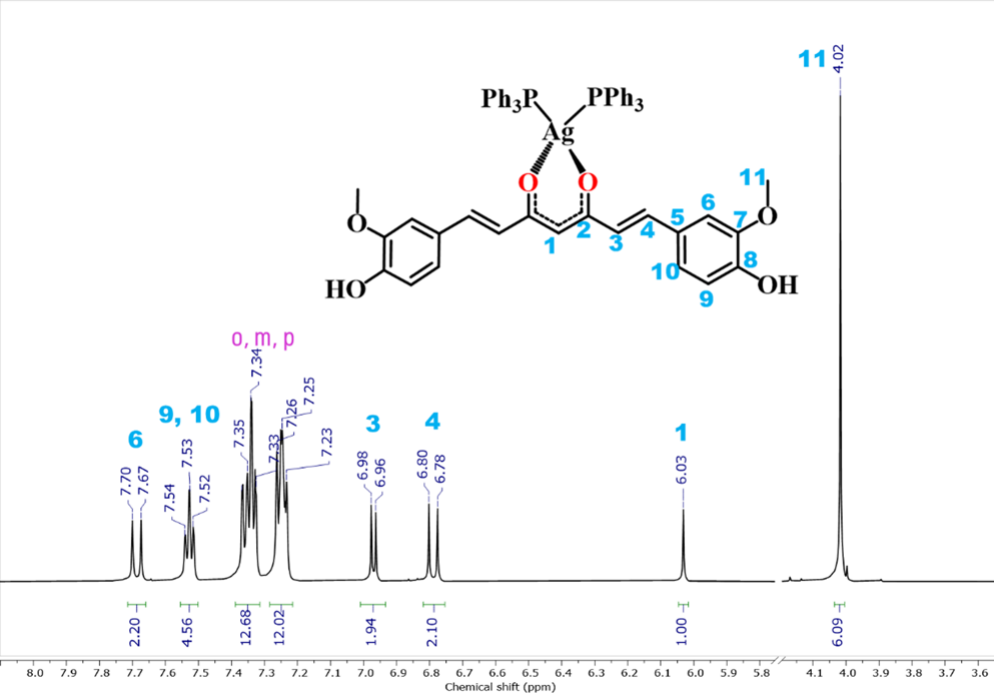
^1^H NMR spectrum of complex **1** (in acetone-d_6_) with hydrogens identified

The ^1^H NMR spectra of the four complexes show the characteristic signals in the aromatic region, corresponding to the hydrogens atoms of the triphenylphosphine and the natural products. All hydrogens of the complexes were assigned and correspond to expected integral, chemical shift and coupling, as shown in the experimental session. Figure 2 shows the ^1^H NMR of complex **1**, where it was possible to identify the methoxy (11), aromatic (6, 9, 10) and vinyl groups (1, 3, 4) of curcumin (in blue), as well as the aromatic (ortho, meta, para) hydrogens of triphenylphosphine (in purple) (numbering scheme of complexes on Fig. 1).

**Fig. 3:**
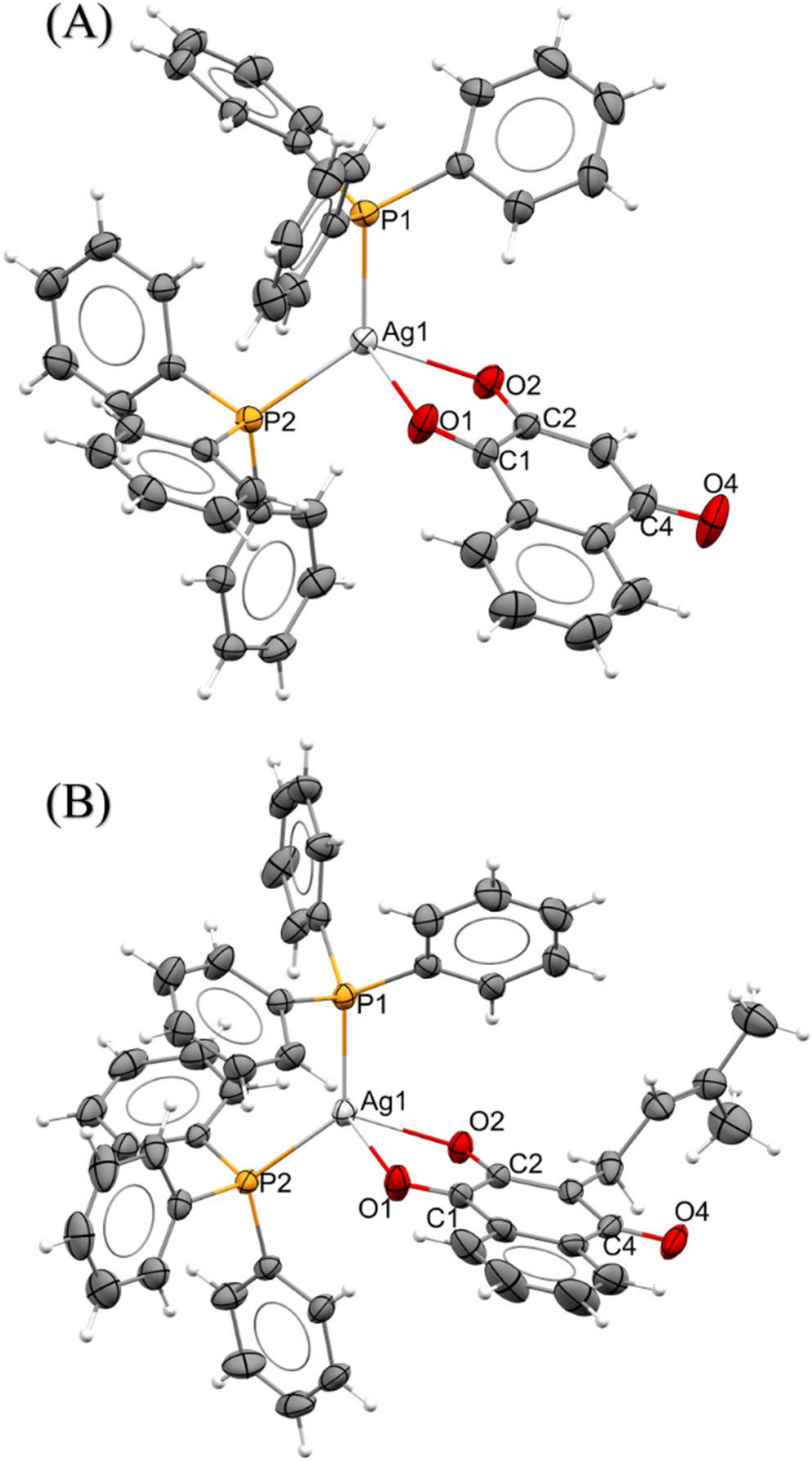
Crystal structures of complexes (**A**) **2** and (**B**) **3**, highlighting selected atom labels and 30% probability ellipsoids

The crystallographic data confirmed the mononuclear structure of the neutral silver complexes. Complexes **2** and **3** crystallize in triclinic and monoclinic space groups, respectively, each containing one molecule per asymmetric unit. The crystal data and structure refinement parameters for complexes 2 and 3 are summarized in Table S1. Figure 3 illustrates the crystal structures of complexes, highlighting selected atom labels and 30% probability ellipsoids. These structures confirm the bidentate coordination (via O, O donor atoms) of the naphthoquinones to the central silver atom, occupying two coordination sites, as depicted. The bond angles P1–Ag1–P2 (126.9° for complex 2 and 130.8° for complex 3) and O1–Ag1–O2 (68.4° for complex 2 and 67.6° for complex 3) are consistent with a distorted tetrahedral coordination environment around the Ag(I) center (Table S2).

The Ag–O bond distances are slightly asymmetric in both structures. For complex 2, Ag1–O1 = 2.5066(14) Å and Ag1–O2 = 2.3036(13) Å, while for complex 3, Ag1–O1 = 2.521(3) Å and Ag1–O2 = 2.297(3) Å (Tables S3 and S4). In both complexes, O1 corresponds to the carbonyl oxygen (C = O), whereas O2 is associated with a C–O single bond. The longer Ag–O1 distances can be attributed to the reduced donor ability of the carbonyl oxygen, whose electron density is partially delocalized within the π-system, while the shorter Ag–O2 bonds reflect the stronger σ-donor character of the C–O oxygen. This asymmetric coordination behavior is consistent with related O, O-chelated silver(I) complexes reported in the literature [27–30]. Additionally, the C–O bond distances closely resemble those of free naphthoquinones, indicating that the electronic delocalization inherent to the molecule remains largely unaffected by coordination.

Full interaction maps (FIMs, Fig. S14) generated using the *Mercury* software provided insights into the intermolecular interaction potential of the studied complexes. The analysis revealed a predominance of hydrophobic regions, highlighted in orange (aromatic CH probe) and red (Carbonyl oxygen probe), which dominate the molecular surface, consistent with its largely nonpolar character. However, a notable exception was observed in the vicinity of the free carbonyl group, which exhibited a distinct hydrophilic blue region (uncharged NH probe). This region is capable of engaging in polar interactions, such as hydrogen bonding, suggesting its critical role in modulating the molecule’s interaction with polar solvents or functional groups in potential biomolecules [31, 32]. 

### Stability studies

**Fig. 4:**
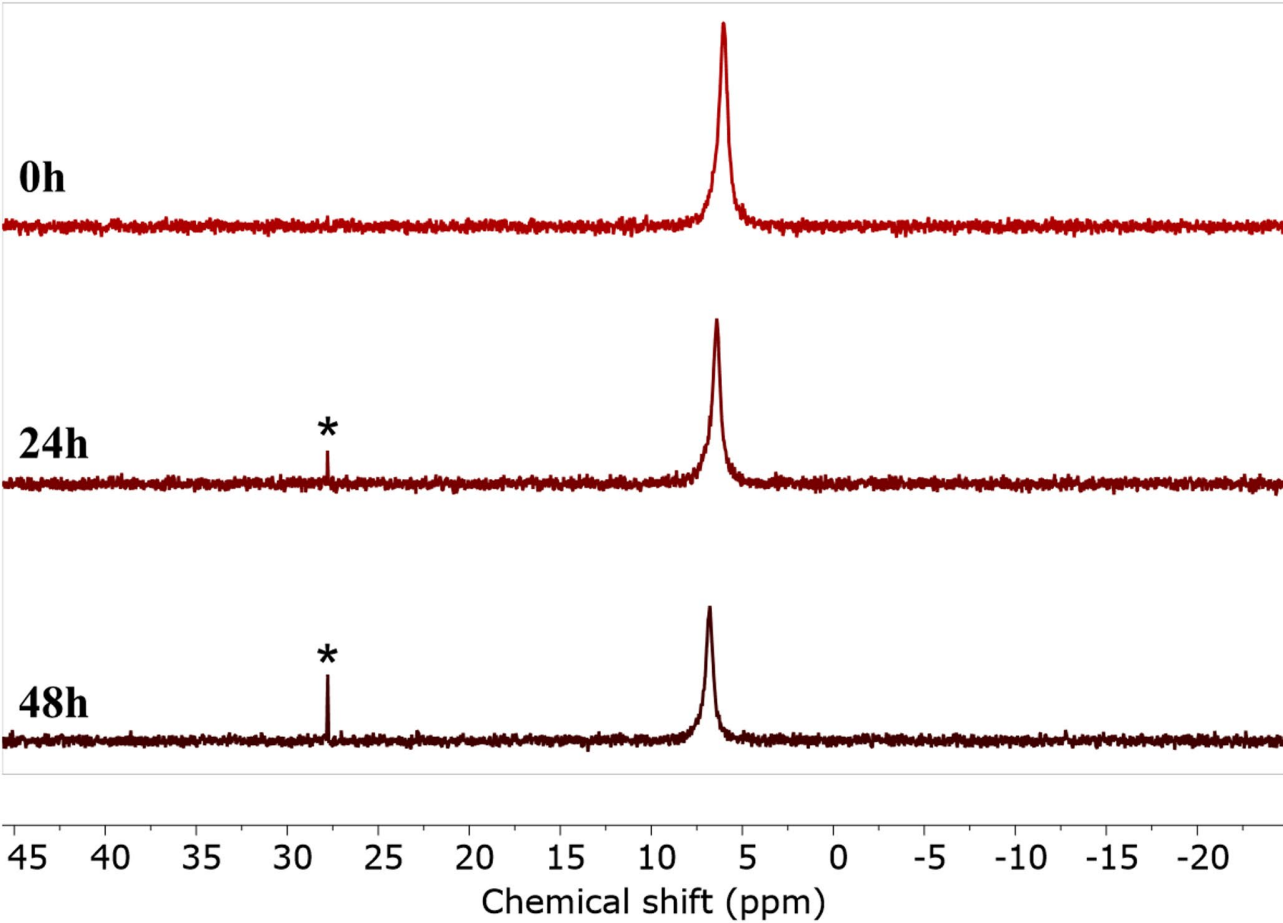
Expansion of ^31^P{^1^H} NMR spectra of complex **1** in DMSO (using a capillary containing D_2_O) recorded at 0, 24 and 48 h after the preparation of the sample. Signals of free PPh_3_ are indicated with *

The behavior of the complexes **1**–**4** in DMSO solution has been studied, in other to investigate the presence of speciation reactions under these conditions. Complex **1** showed a ^31^P{^1^H} NMR signal of approximately 30 ppm after 24 h in solution (as indicated with * in Fig. 4) corresponding to free PPh_3_. This indicates that complex **1** has a small dissociation of the phosphine ligand in this solvent, however a large part of the complex is preserved up to 48 h. Complexes **2**–**4** were stable in DMSO solution for up to 48 h as demonstrated in ^31^P{^1^H} NMR spectra (see Electronic Supporting Information, Fig. S15).

### Cell viability in vitro

The in vitro cell viability of the compounds were determined against cell lines of human tumor and non-tumor using the colorimetric MTT method after 48 h of incubation. The complexes performed strong cytotoxicities against all cell lines tested, and the IC_50_ values and the selective index were listed in Table 2. Cisplatin was used as standard for comparison.

The synthesized complexes were more active than cisplatin in breast, lung and ovarian tumor cell lines, highlighting the lower IC_50_ found in ovarian cells. These results are in line with the literature, which demonstrates that the coordination of natural products to metal centers enhances biological activity, including antitumor activity [13, 33–35]. This approach may also be promising in the search for new therapeutic strategies.

The Ag(I) compounds exhibited less selective index (SI) values compared to cisplatin in the MCF-7 and A549 cell lines (Table 2). Among the complexes, complex **2** was found oud as most selective index against MDA-MB-231 (3.0), but this SI was smaller than that of the precursor (3.9). On the other hand, complex **2** exhibited higher SI value (2.1) compared to precursor and cisplatin (0.9) in A2780cis cell line. It is crucial to highlight that silver compounds can undergo competing ligand exchange reactions with biological components during cytotoxic experiments, potentially forming new species that may account for the observed activity, as demonstrated in other works in the literature [15, 16]. 

**Table 2:** IC_50_ values (µM) of complexes, ligands and cisplatin against MDA-MB-231, MCF-7, MCF-10 A, A549, A2780cis and MRC-5 cell lines at 48 h

	IC_50_ (µM)						Selective Index (SI)			
	MDA-MB-231	MCF-7	MCF-10 A	A549	A2780cis	MRC-5	SI^b^	SI^c^	SI^d^	SI^e^
**1**	2.5 ± 0.3	5.6 ± 0.3	3.9 ± 0.2	9.1 ± 0.5	0.8 ± 0.1	1.3 ± 0.2	1.5	0.7	0.1	1.6
**2**	1.8 ± 0.3	4.6 ± 0.2	5.4 ± 0.3	1.9 ± 0.3	1.4 ± 0.2	3.0 ± 0.3	3.0	1.2	1.6	2.1
**3**	2.6 ± 0.6	14.2 ± 0.7	3.5 ± 0.4	3.0 ± 0.1	1.3 ± 0.1	1.2 ± 0.2	1.6	0.2	0.4	0.9
**4**	4.1 ± 0.5	5.9 ± 0.5	5.2 ± 0.3	3.5 ± 0.2	1.4 ± 0.2	1.6 ± 0.3	1.3	0.9	0.5	1.1
Prec^*a*^	1.9 ± 0.2	8.8 ± 0.6	7.4 ± 0.2	3.2 ± 0.2	1.2 ± 0.2	1.1 ± 0.1	3.9	0.8	0.3	0.9
Cur	40.6 ± 0.5	36.8 ± 1.6	-	38.9 ± 1.3	22.8 ± 2.5	17.4 ± 0.2	-	-	-	-
Ali	≥ 100	≥ 100	≥ 100	≥ 100	36.0 ± 0.8	≥ 100	-	-	-	-
CP	10.2 ± 0.2 [36]	8.6 ± 1.8	29.5 ± 0.8	14.4 ± 1.4	31.1 ± 1.8	29.1 ± 0.8	2.9	3.4	2.0	0.9

Cur = curcumin; ali = alizarin; CP = cisplatin; ^*a*^[Ag(PPh_3_)_2_CH_3_CN]ClO_4_; ^*b*^IC_50_ MCF-10 A/ IC_50_ MDA-MB-231; ^*c*^IC_50_ MCF-10 A/ IC_50_ MCF-7IC_50_; ^*d*^IC_50_ MRC-5/ IC_50_ A549; ^*e*^IC_50_ MRC-5/ IC_50_ A2780cis. IC_50_ for ligands PPh_3_, lawsone and lapachol ≥ 100 µM

### Anti-*mycobacterium tuberculosis* activity

The precursor, rifampicin, *natural products* and its Ag(I) complexes have been evaluated against *M. tuberculosis* H37Rv and the results are listed in Table 3. The compounds were tested starting from a concentration of 25 µg/mL and the ideal concentration to approve a substance is ≤ 10 µg/ml, that is, the substances must be able to inhibit 90% or more of the growth of *Mycobacterium tuberculosis* with such concentration in the medium [37, 38]. The results were expressed as MIC_90_ values (µg/mL) the lowest concentration resulting in 90% inhibition of growth.

All synthesized complexes showed better activity against Mtb than the free ligands and the precursor. Complex 4 showed the best result, among the synthesized complexes, with MIC_90_ of 0.82 ± 0.10 µg/mL and alizarin showed a good result, with MIC_90_ of 1.00 ± 0.52 µg/mL. Furthermore, complex 4 presented better SI, as well as its free ligand, alizarin. However in the literature we observed that Ag(I) complexes with N -acylhydrazones were more active and selective for the same purpose [39]. 

**Table 3:** MIC_90_ values determined for the precursors, ligands and Ag(I) complexes against *Mycobacterium tuberculosis* (H_37_Rv). MRC-5 cells assays and SI

Compounds	MW (g/mol)	MIC_90_ (µg/mL)	MIC_90_ (µmol/L)	IC_50_ (µmol/L)	SI
**1**	999.82	2.36 ± 0.40	2.37	1.3 ± 0.2	-
**2**	805.59	2.44 ± 0.16	3.03	3.0 ± 0.3	-
**3**	873.71	1.43 ± 0.12	1.64	1.2 ± 0.2	-
**4**	871.65	0.82 ± 0.10	0.94	1.6 ± 0.3	1.7
cur	368.38	5.78 ± 1.16	15.71	17.4 ± 0.2	1.1
law	174.15	> 25	-	≥ 100	-
lap	242.27	22.00 ± 0.83	90.84	≥ 100	1.1
ali	240.20	1.00 ± 0.52	4.18	≥ 100	23.9
PPh_3_	262.29	> 25	-	≥ 100	-
Precursor	772.95	12.55 ± 2.70	16.24	1.1 ± 0.1	-
Rifampicin	822.94	0.04 ± 0.01	0.06	-	-

MW = Molar Weight

### Multidrug-resistant clinical strains of *M. tuberculosis*

The emergence of multidrug-resistant (MDR) strains of *Mycobacterium tuberculosis* (Mtb) poses a significant challenge to global health, necessitating the exploration of novel therapeutic agents. Recent studies have highlighted the potential of silver complexes as effective antimicrobial agents against MDR Mtb strains [40, 41]. 

The low MIC_90_ values of complex 4, described in Table 3, encouraged us to evaluate this compound against 5 clinical strains. The clinical isolates (CIs) of Mtb tested in this study exhibited resistance to multiple first-line anti-TB drugs, including isoniazid (INH) and rifampicin (RIF), as well as to the second-line drugs linezolid (LNZ) and moxifloxacin (MOX). Specifically, CI-1 and CI-3 demonstrated resistance to LNZ, INH and RIF, while CI-2, CI-4 and CI-5 were resistant to INH alone [40, 41]. 

Complex **4** also showed good activity against 4 of the 5 MDR Mtb strains tested, as shown in Table 4. This finding aligns with the broader literature indicating that silver nanoparticles (AgNPs) exhibit promising antibacterial properties against various drug-resistant bacteria, including Mtb [42, 43]. The ability of silver complexes to maintain efficacy against these resistant strains is particularly noteworthy, as the rise of MDR-TB has been attributed to inadequate treatment regimens and the misuse of existing antibiotics [44, 45]. 

The in vitro evaluation of silver complex against these resistant strains suggests a potential role for silver-based therapies in the treatment of MDR-TB, as supported by previous research indicating that biosynthesized silver nanoparticles possess anti-mycobacterial activity against MDR strains [40, 46]. 

**Table 4:** In vitro activity against clinical isolates of Mtb

Compounds	MIC_90_ (µg/mL)					
	Complex 4	LNZ	MOX	INH	RIF	
**CI-1**	3.82 ± 0.61	> 10	4.66 ± 0.29	> 10	> 10	
**CI-2**	3.01 ± 0.36	0.14 ± 0.01	2.43 ± 0.06	> 10	0.82 ± 0.32	
**CI-3**	> 10	> 10	4.93 ± 0.08	> 10	> 10	
**CI-4**	1.97 ± 0.62	0.42 ± 0.16	1.16 ± 0.01	> 10	> 10	
**CI-5**	4.80 ± 0.25	1.38 ± 0.74	1.48 ± 0.43	> 10	0.86 ± 0.39	
**H**_**37**_ **Rv**	0.821	0.110	0.040	0.230	0.004	

CI: Clinical Isolates; LNZ: linezolid; MOX: moxifloxacin; INH: isoniazid and RIF: rifampicin; recorded data are the average of the results from two or three independent assays

Moreover, the mechanism of action of silver complexes against Mtb is an area of ongoing investigation. Studies have suggested that silver ions can disrupt bacterial cell membranes, leading to increased permeability and eventual cell death [43]. Additionally, the interaction of silver with bacterial proteins may inhibit essential metabolic processes, further contributing to its antimicrobial efficacy [42, 43]. However, the precise mechanisms by which silver complexes exert their effects on Mtb require further elucidation, as highlighted by Sadhu et al., who called for more detailed research into the action of silver nanoparticles against tuberculosis [41]. 

In conclusion, the promising results obtained from the evaluation of silver complexes against clinical isolates of Mtb underscore their potential as alternative therapeutic agents in the fight against drug-resistant tuberculosis. The ability of these compounds to inhibit the growth of MDR strains at low concentrations is particularly significant, given the urgent need for new treatments in the context of rising antibiotic resistance. Future studies should focus on optimizing the formulation and delivery of silver complexes, as well as elucidating their mechanisms of action, to enhance their therapeutic potential against Mtb.

## Conclusions

New Ag(I) complexes with natural products were obtained. All complexes were analyzed by analytical methods and spectroscopic techniques such as ^1^H, ^13^C and ^31^P{^1^H} NMR, IR, elemental analysis and molar conductivity. Crystal structure of complexes **2** and **3** was solved and confirmed the purpose structure, as well as the other techniques used. The Ag(I) coordination of all synthesized complexes occurs by O atoms of the natural products deprotonated, by bidentate form. All synthesized silver complexes showed lower IC_50_ than cisplatin and free ligands, showing that this was a good strategy for obtaining active metal complexes. However, the synthesized complexes did not show good selectivity, as they were also very active in normal cells. Biological tests in vitro were performed for all compounds synthesized, ligands and precursor against *M. tuberculosis* H37Rv. By means of the MIC values, it can be stated that all tested compounds showed anti- *M. tuberculosis* activities, including free alizarin. Complex **4** showed the best result with MIC_90_ of 0.821 ± 0.108 µg/mL. Indeed, the results of the assays over 5 clinical *M. tuberculosis* multidrug-resistant strains evidenced that the complex **4** showed activity for clinical strains 1, 2, 4 and 5, with MIC values > 5 µg/mL. Silver complexes show promise as alternative treatments for drug-resistant tuberculosis (MDR-TB), effectively inhibiting MDR strains at low concentrations. Further research should optimize their formulation, delivery, and mechanisms of action to maximize their therapeutic potential.

## Supplementary Information

Below is the link to the electronic supplementary material.

Supplementary Material 1

Supplementary Material 2

Supplementary Material 3

Supplementary Material 4

Supplementary Material 5

## Data Availability

No datasets were generated or analysed during the current study.
