# Supplementary material for: Ag(I) complexes with natural products and triphenylphosphine: activity against tumor cell lines and mycobacterium tuberculosis
Source: J Biol Inorg Chem. 2026 Apr 28;31(3):211–21. doi: 10.1007/s00775-026-02144-1 (PMC13287109; doi:10.1007/s00775-026-02144-1)
Supplement: Supplementary file 1 — Supplementary Material 1 [file 775_2026_2144_MOESM1_ESM.docx]

**Electronic Supplementary Information (ESI)**

**Ag(I) complexes with natural products and triphenylphosphine: activity against tumor cell lines and *mycobacterium* *tuberculosis***

Jocely Lucena Dutra^a,b*^, João Honorato de Araujo-Neto^c^, Rafael Wendel Rodrigues Santana^a^, Rone Aparecido De Grandis^d^, Giulia Polinário^d^, Fernando Rogério Pavan^d^, Javier Ellena^e^, Alzir Azevedo Batista^a^*.

*^a^ Departament of Chemistry, Federal University of São Carlos (UFSCar), CEP 13561-905, São Carlos, SP, Brazil*

*^b^ Departament of Chemistry, Federal University of Amazonas (UFAM), CEP 69077-000, Itacoatiara, AM, Brazil*

*^c^ Department of Fundamental Chemistry, Institute of Chemistry, University of São Paulo (USP), CEP 05508-000, São Paulo, SP, Brazil*

*^d^ Department of Biological Sciences, School of Pharmaceutical Sciences of São Paulo State University (UNESP), CEP 14800-903, Araraquara, SP, Brazil.*

*^e^ São Carlos Institute of Physics, University of São Paulo (USP), CP 369, CEP 13560-970, São Carlos, SP, Brazil.*

***Corresponding Author:** Jocely Lucena Dutra and Alzir A. Batista
 E-mail: [jocely.dut@hotmail.com](mailto:jocely.dut@hotmail.com); [daab@ufscar.br](mailto:daab@ufscar.br)

|  |
| --- |

**Fig. S1**. IR spectra of complexes **1-4**.

**Fig. S2.** IR spectrum of curcumin.

**Fig. S3.** IR spectrum of lawsone.

**Fig. S4.** IR spectrum of lapachol.

**Fig. S5.** IR spectrum of alizarin.

**Fig. S6.** ^31^P NMR spectra of [Ag(PPh_3_)_2_CH_3_CN]ClO_4_, in CH_2_Cl_2_ using a capillary containing D_2_O.

***Fig. S7.*** ^31^P NMR spectra of triphenylphosphine, in CH_2_Cl_2_ using a capillary containing D_2_O.


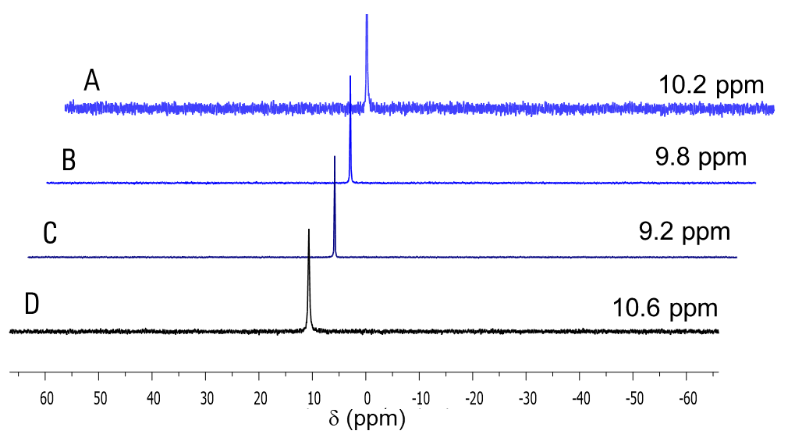


**Fig. S8.** ^31^P NMR spectra of complexes (A) **1**; (B) **2**; (C) **3** and (D) **4**, in CH_2_Cl_2_ using a capillary containing D_2_O.

| 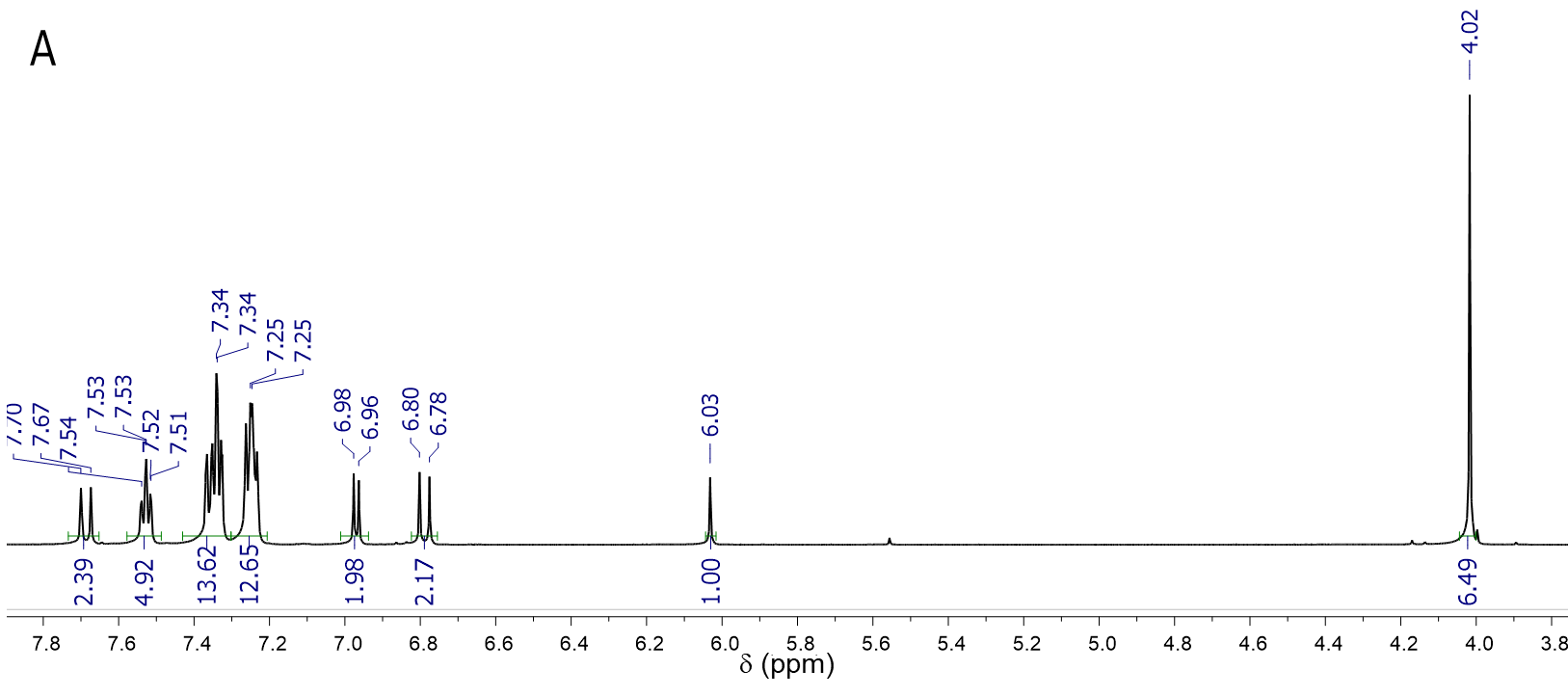 |
| --- |
| 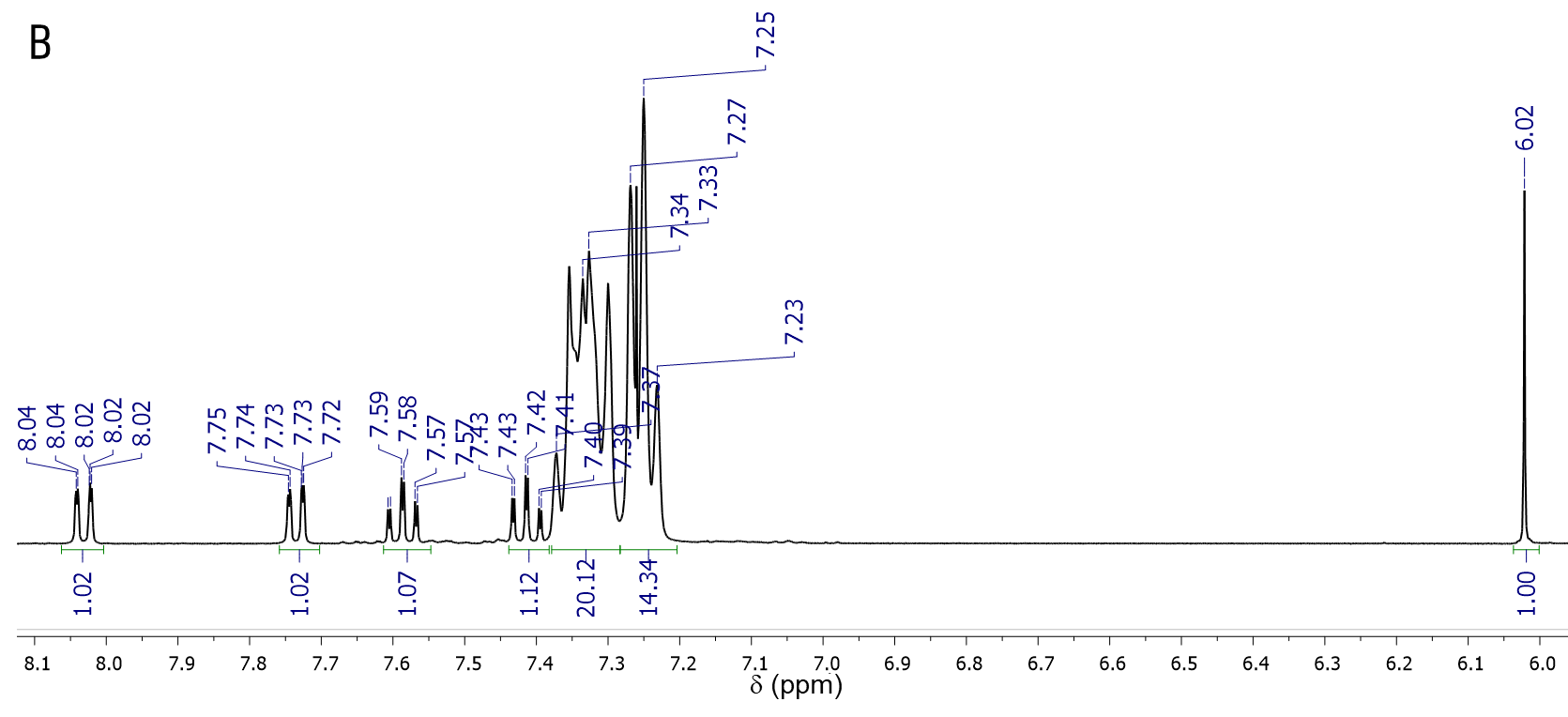 |
| 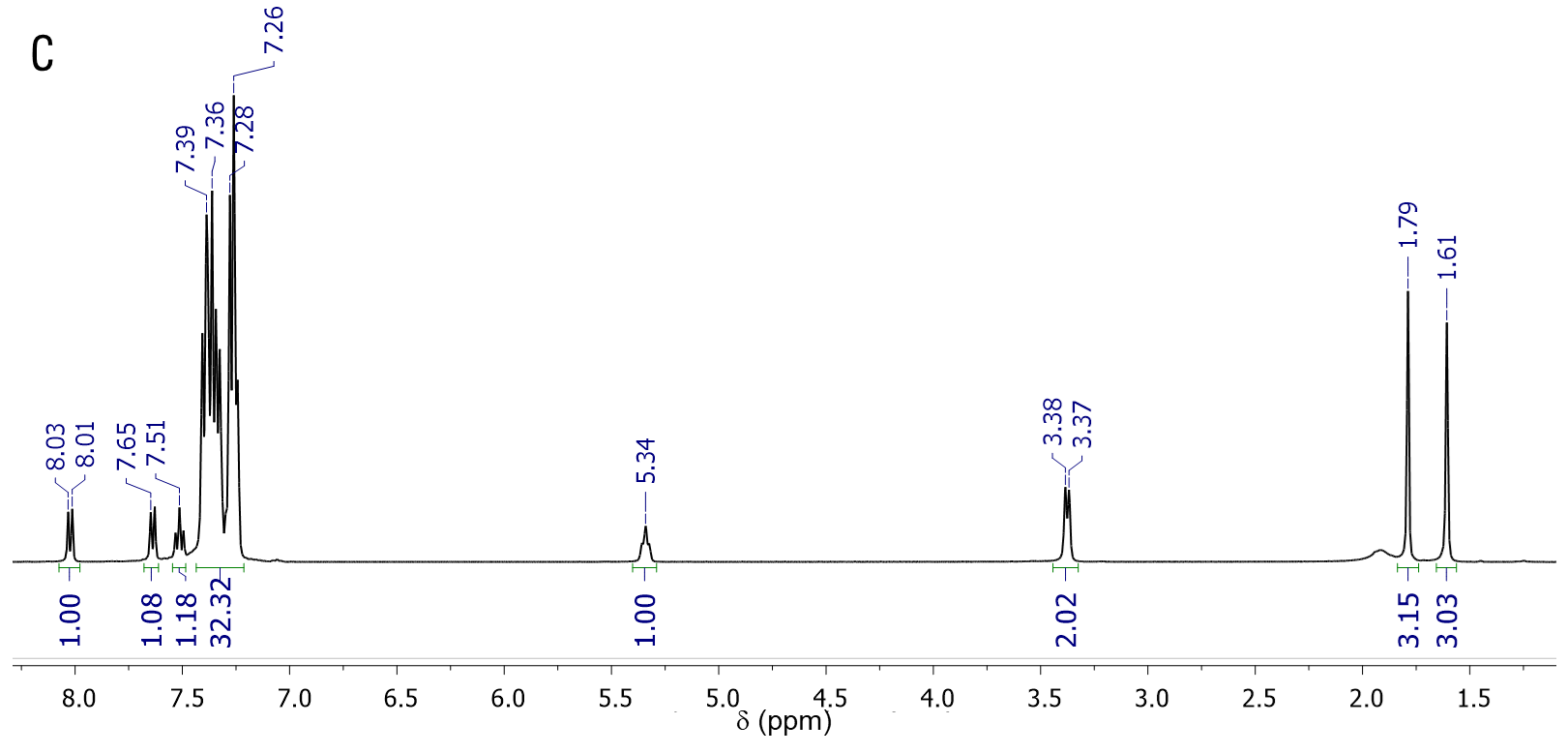 |
| 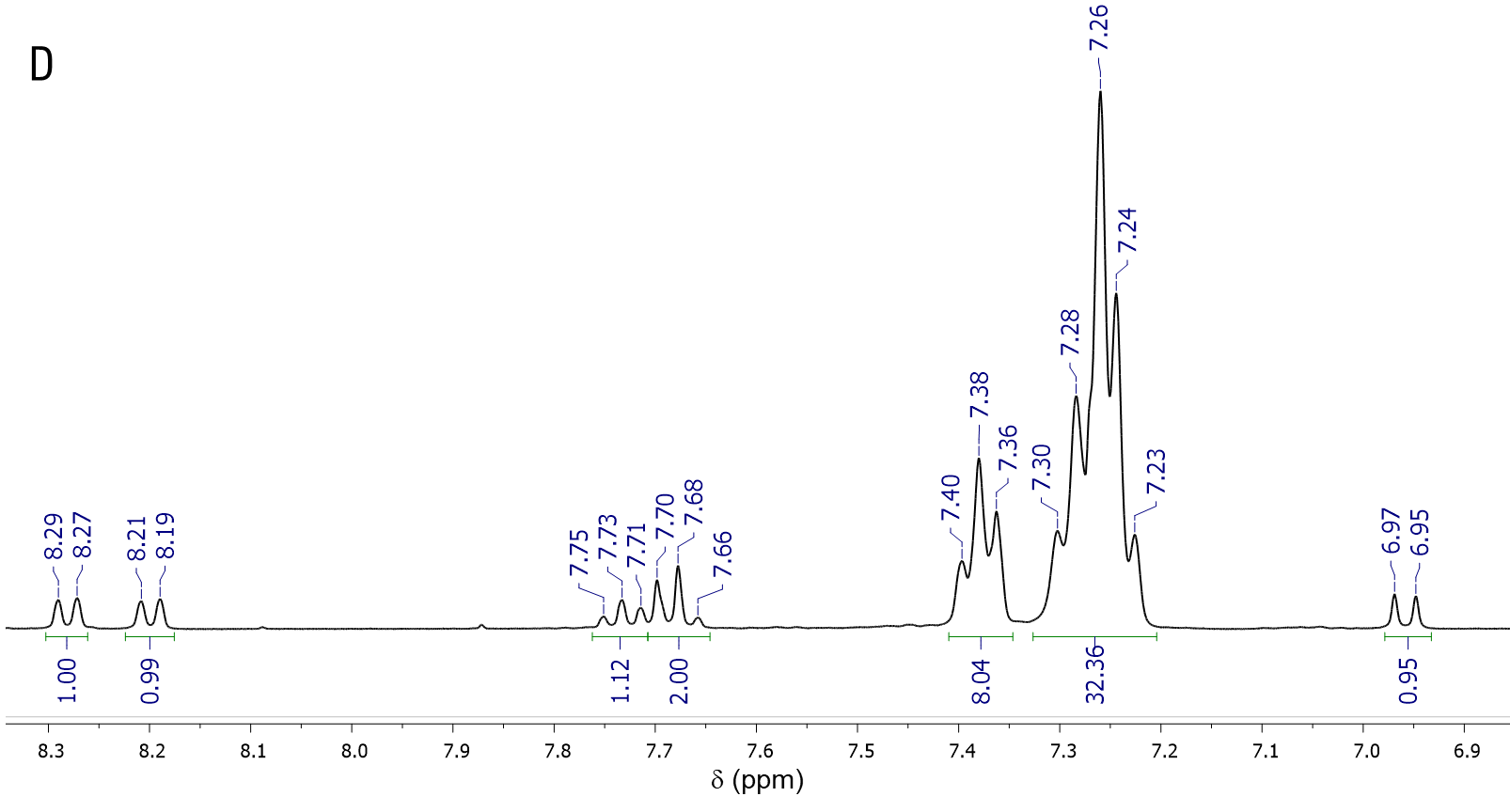 |

**Fig. S9.** ^1^H NMR spectra of complexes (A) **1**; (B) **2**; (C) **3** and (D) **4**, in acetone-d_6_.

| 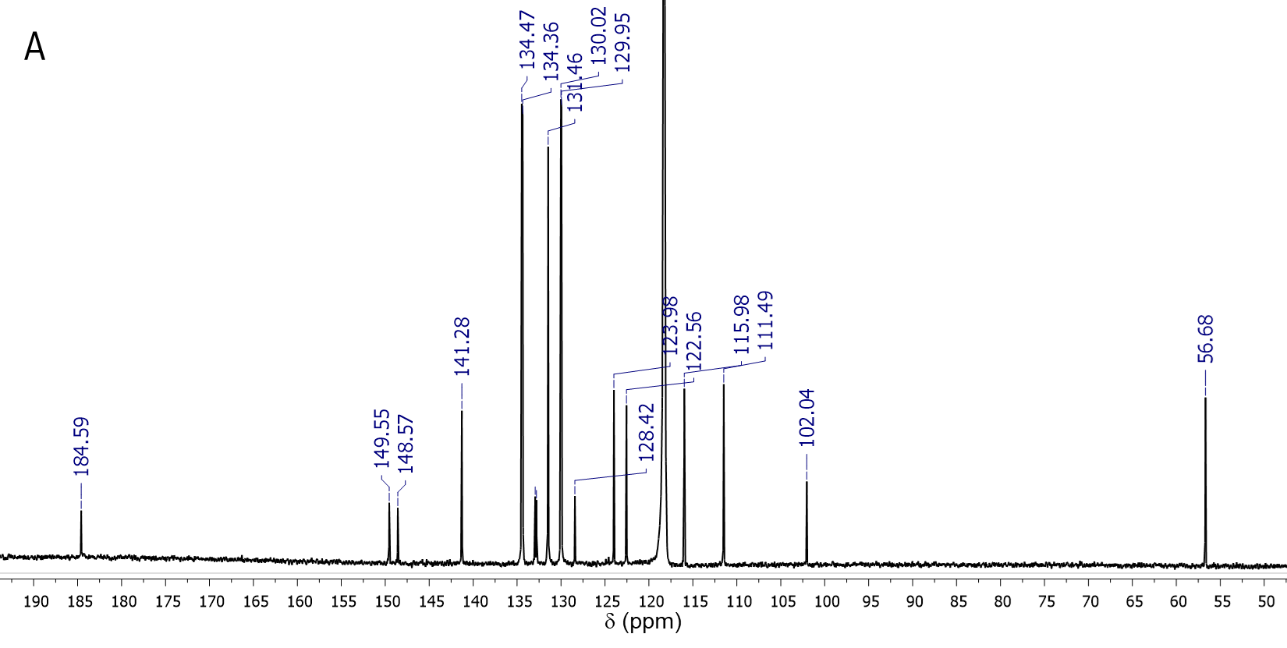 |
| --- |
| 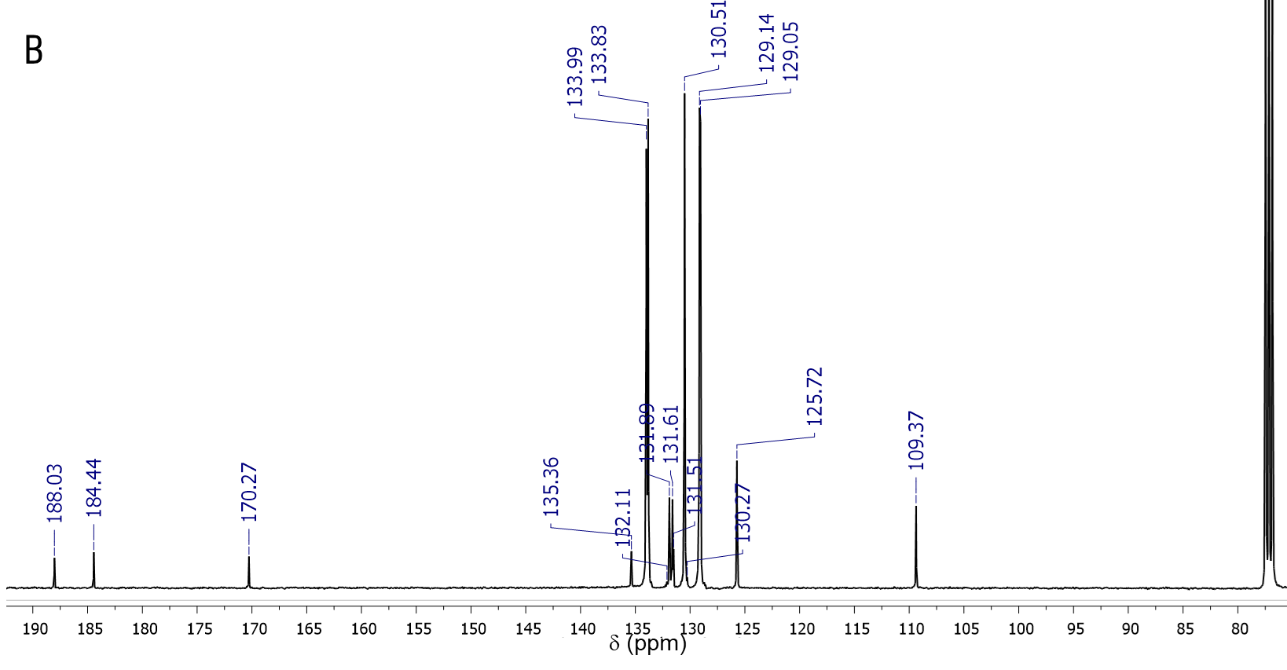 |
| 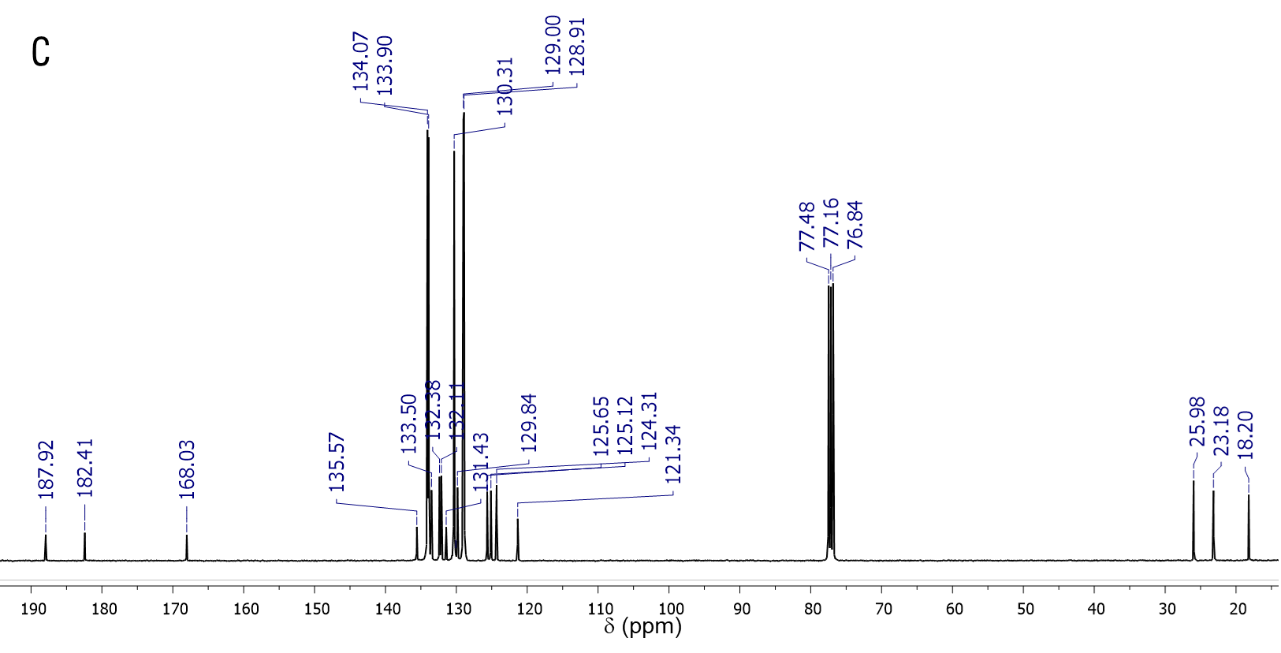 |
| 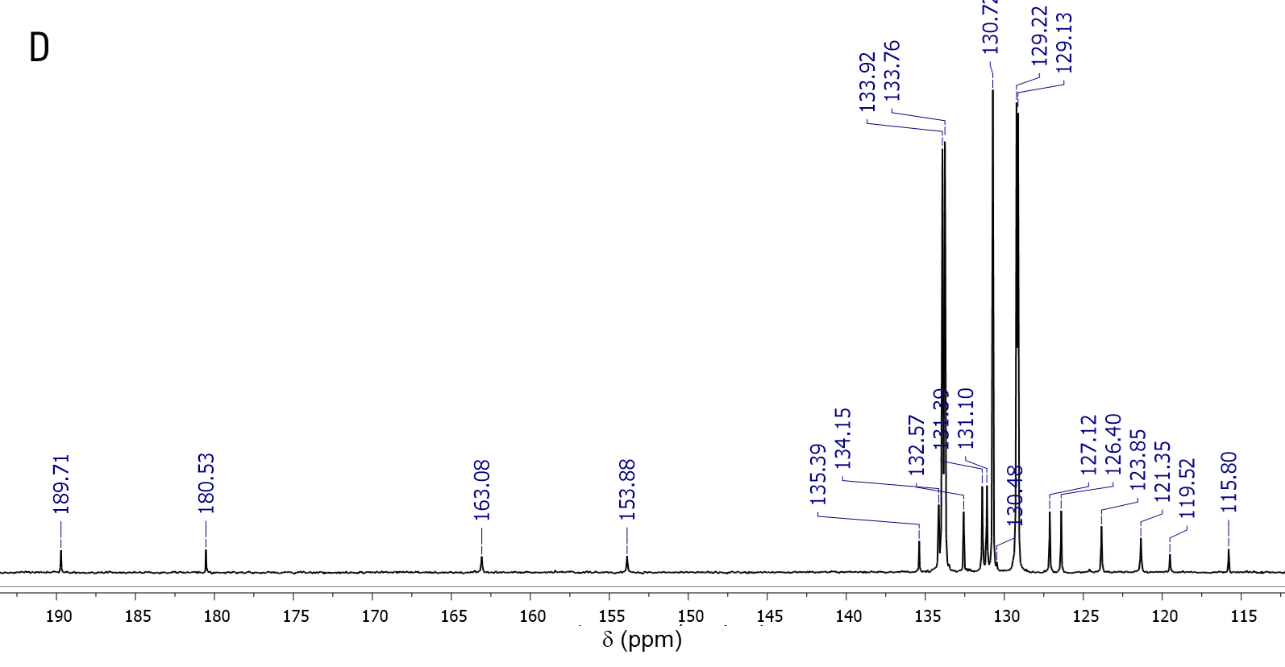 |

**Fig. S10.**  ^13^C NMR spectra of complexes (A) **1**; (B) **2**; (C) **3** and (D) **4**, in acetone-d_6_.

| 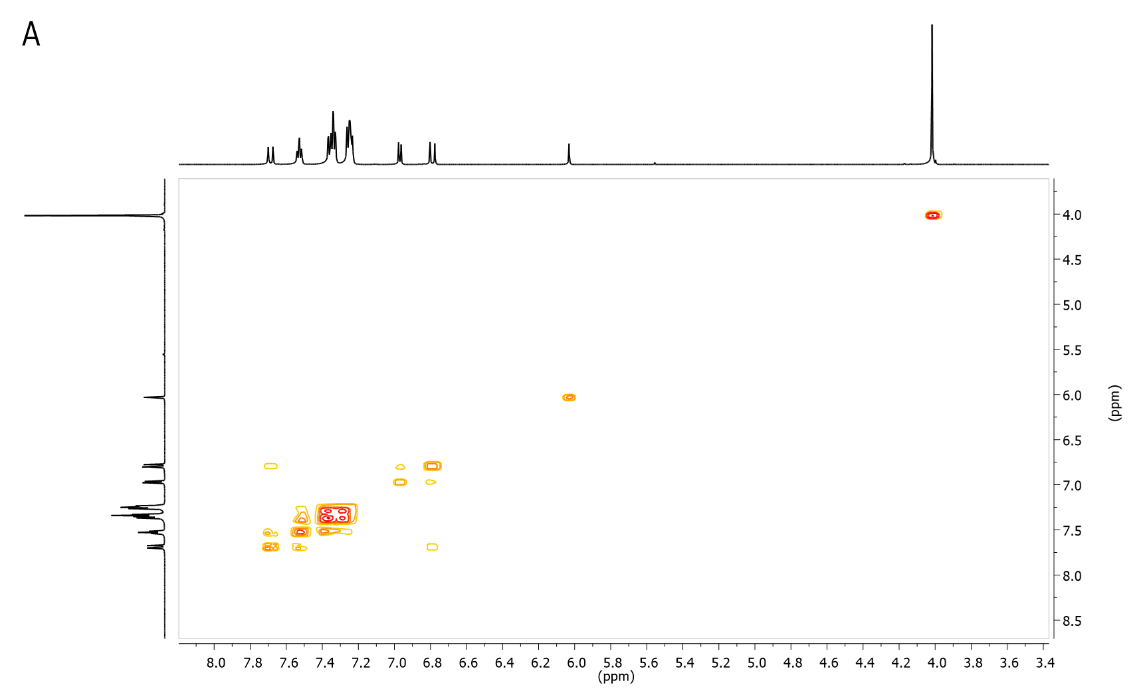 |
| --- |
| 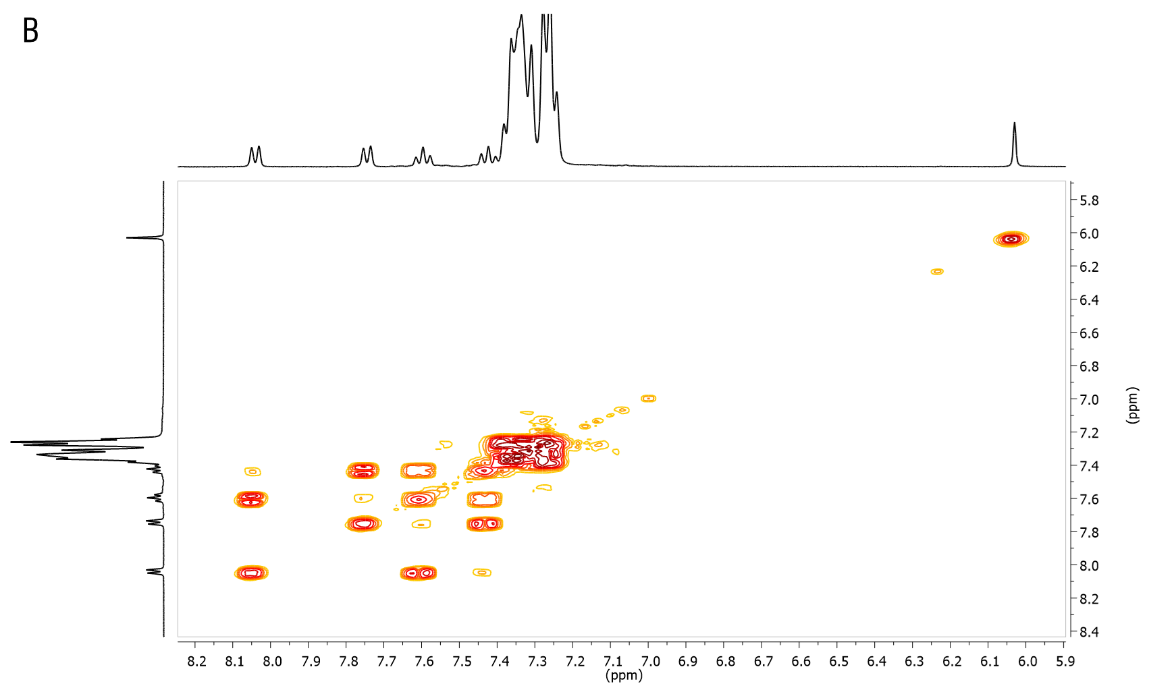 |
| 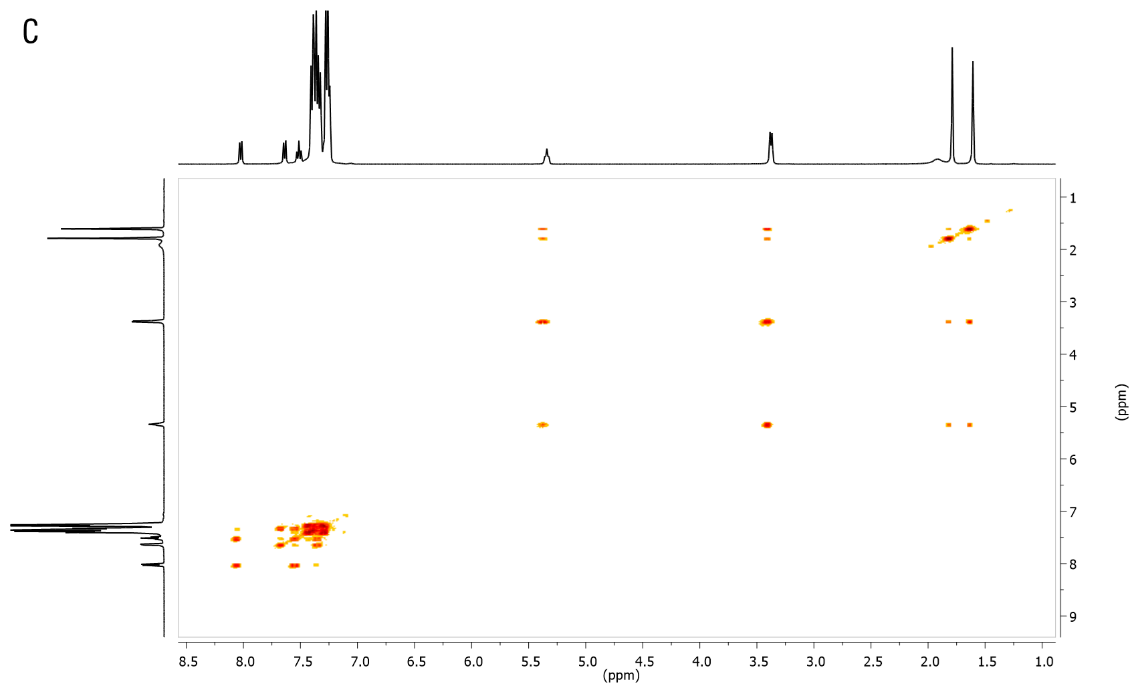 |
| 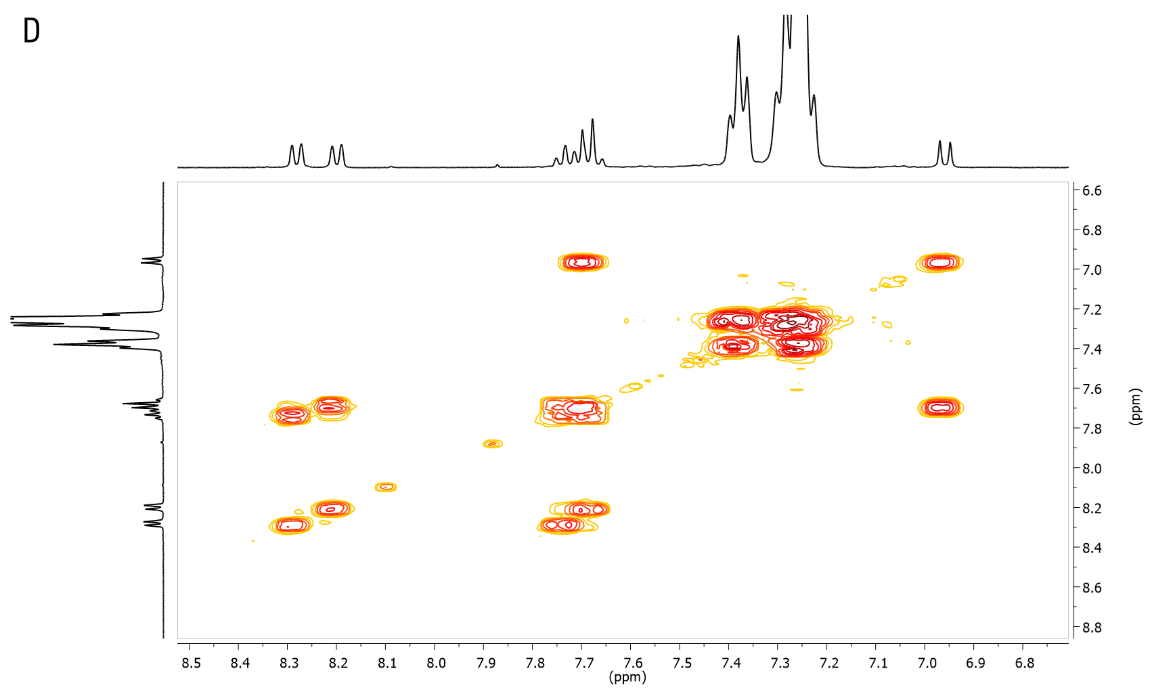 |

**Fig. S11.** COSY ^1^H-^1^H NMR spectra of complexes (A) **1**; (B) **2**; (C) **3** and (D) **4**, in acetone-d_6_.

| 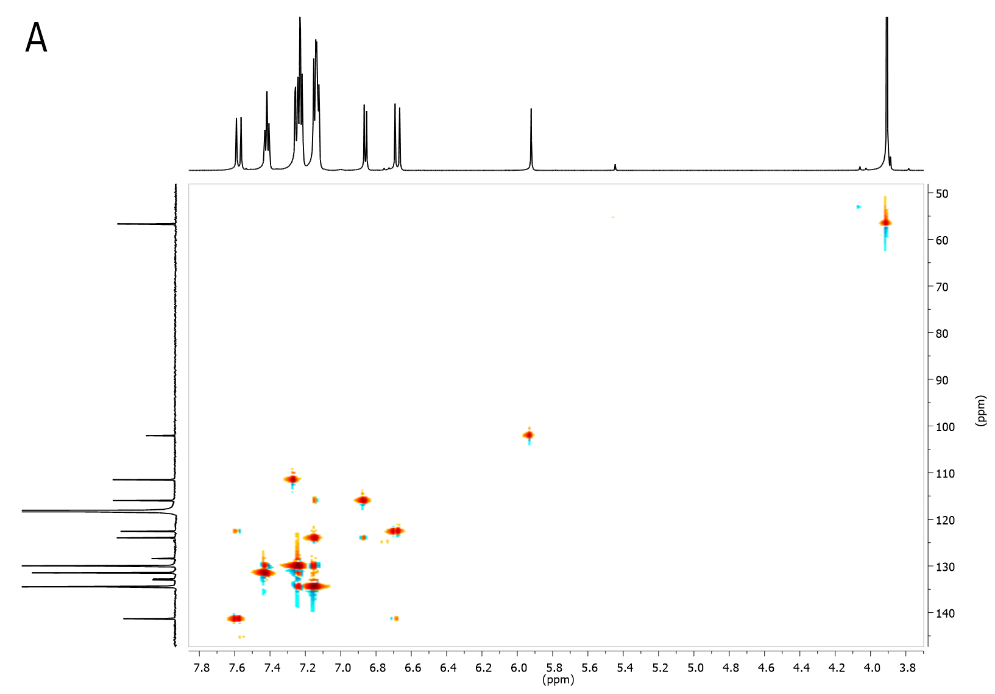 |
| --- |
| 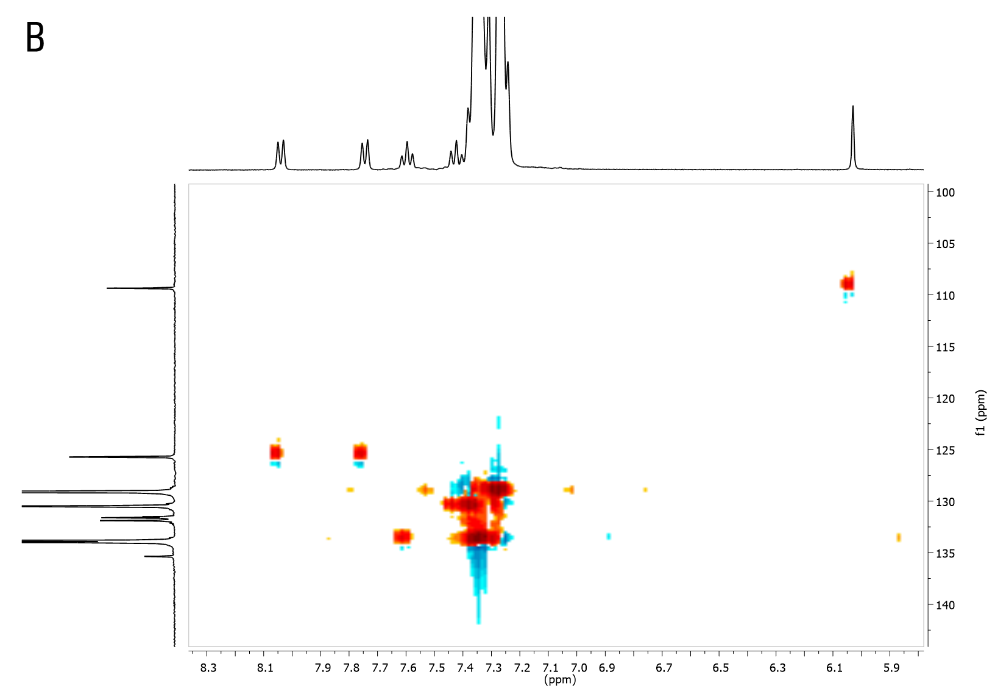 |
| 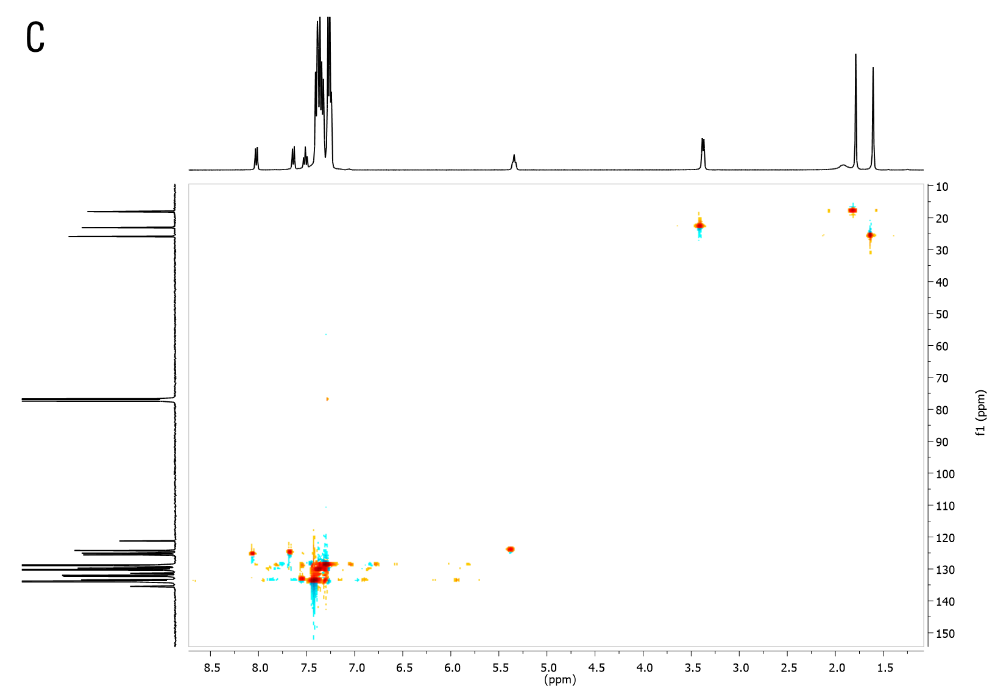 |
| 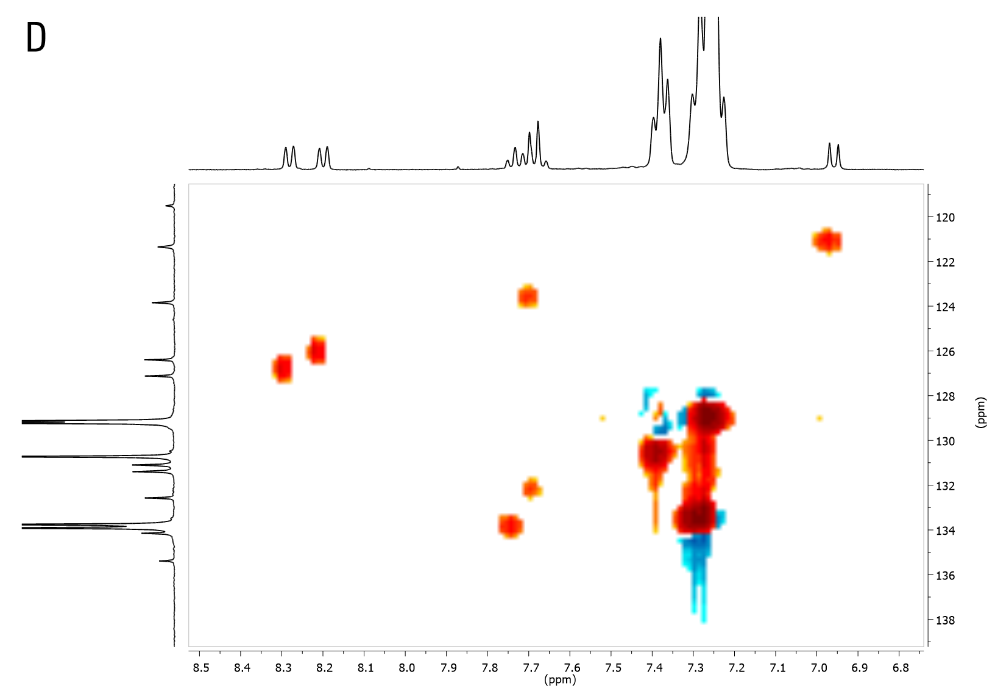 |

**Fig. S12**. ^1^H-^13^C HSQC spectra of complexes (A) **1**; (B) **2**; (C) **3** and (D) **4**, in acetone-d_6_.

| 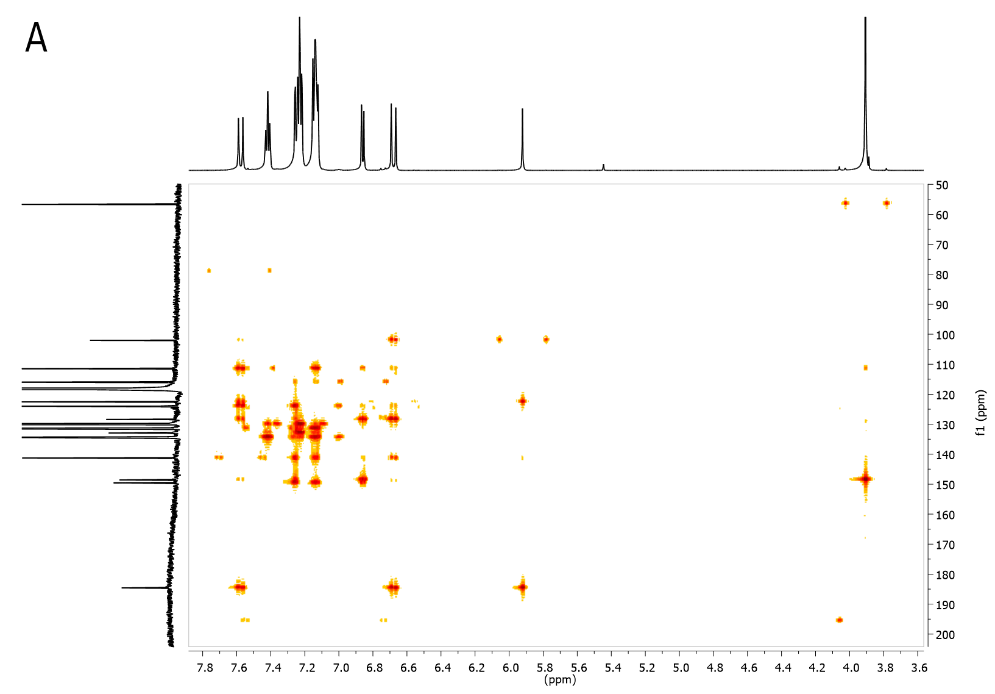 |
| --- |
| 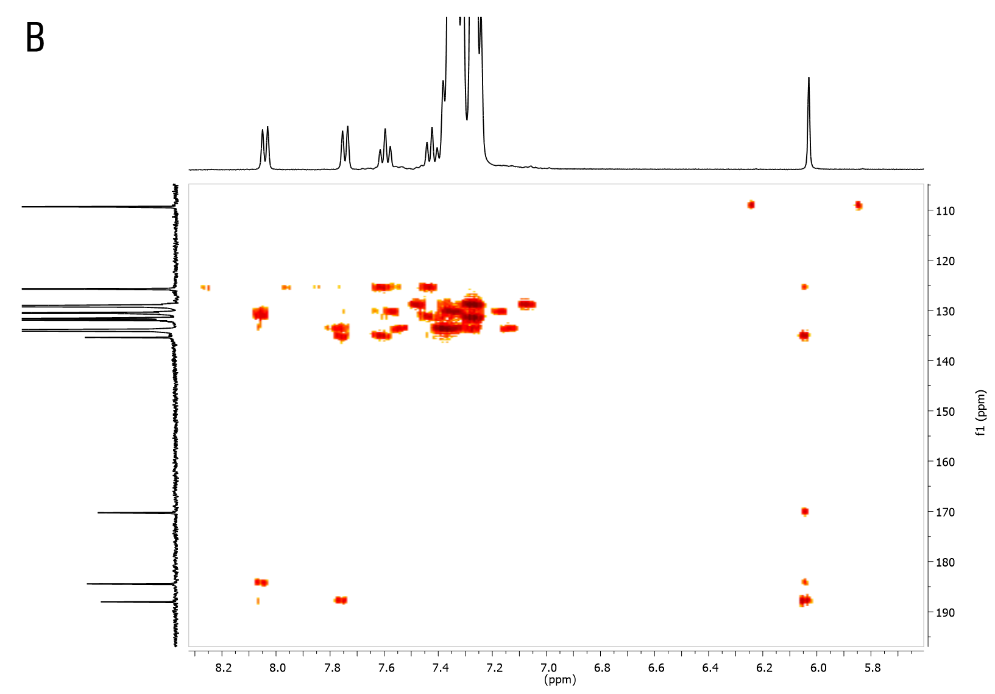 |
| 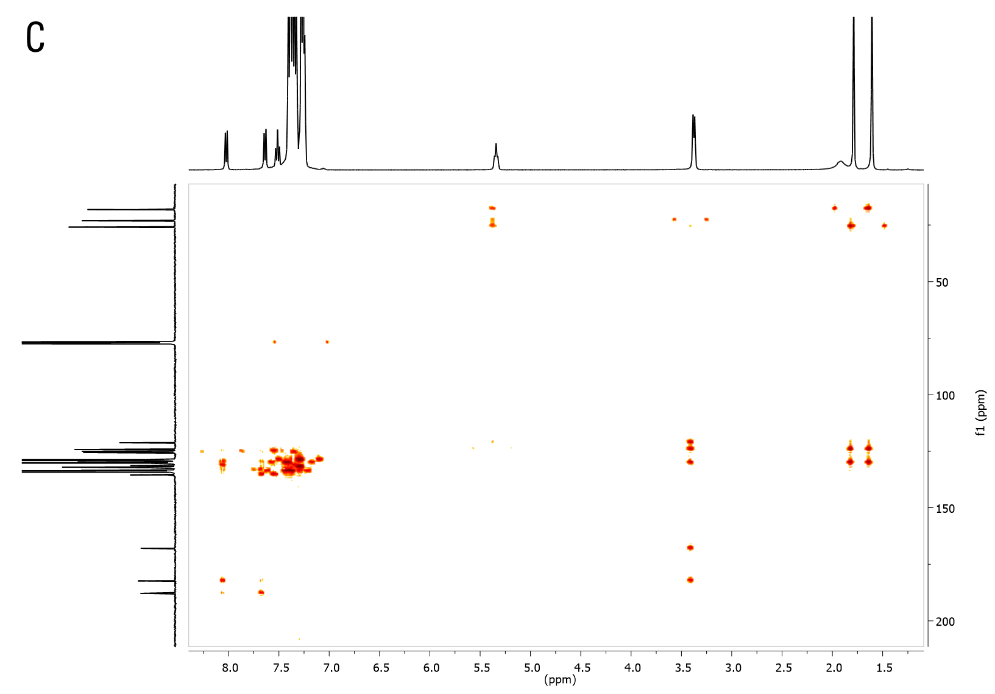 |
| 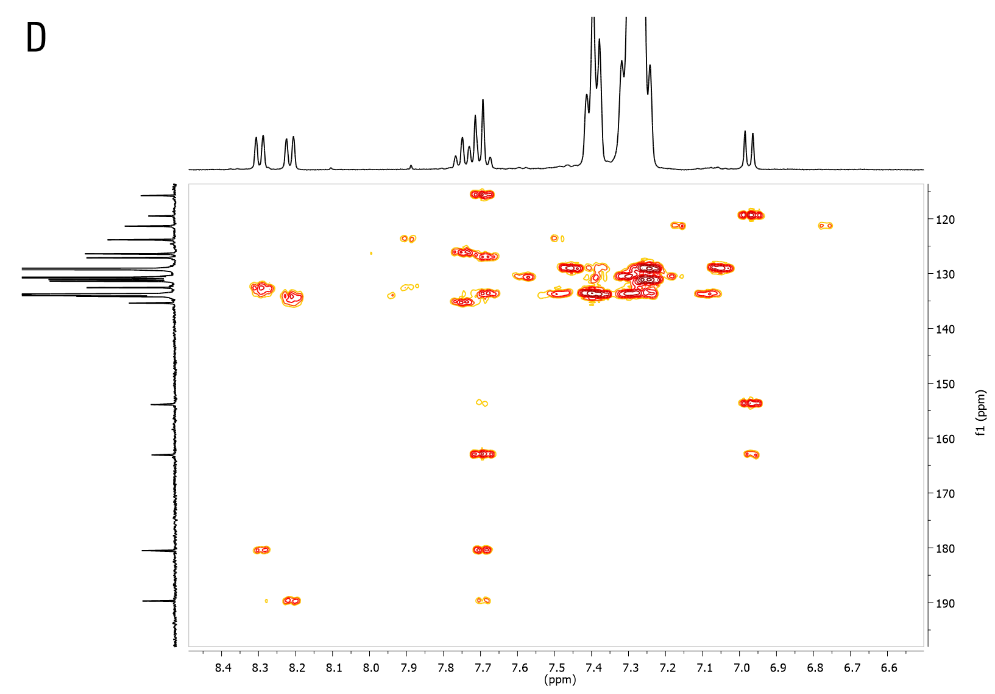 |

**Fig. S13.** ^1^H-^13^C HMBC spectra of complexes (A) **1**; (B) **2**; (C) **3** and (D) **4**, in acetone-d_6_.


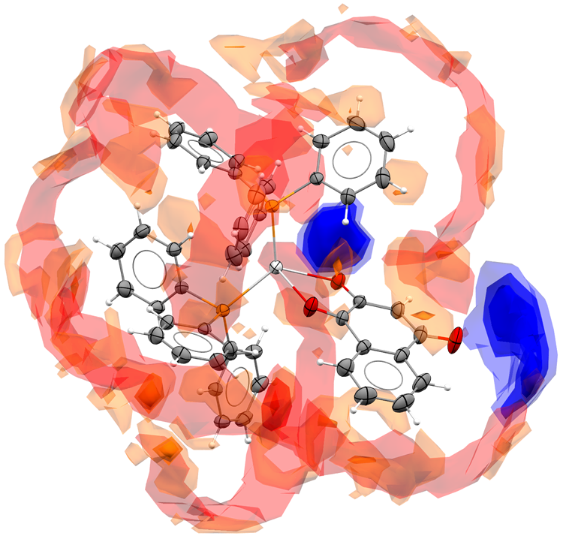


**Fig. S14.** Full Interaction Map generated for complex **3**.

| 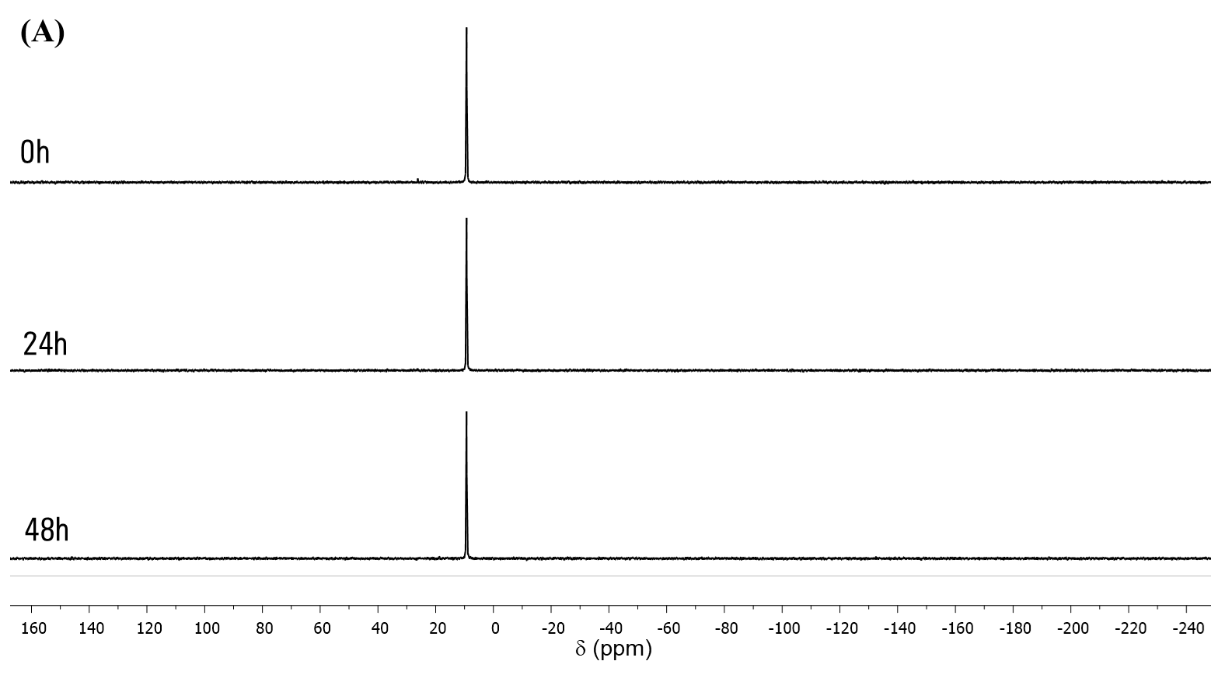 |
| --- |
| 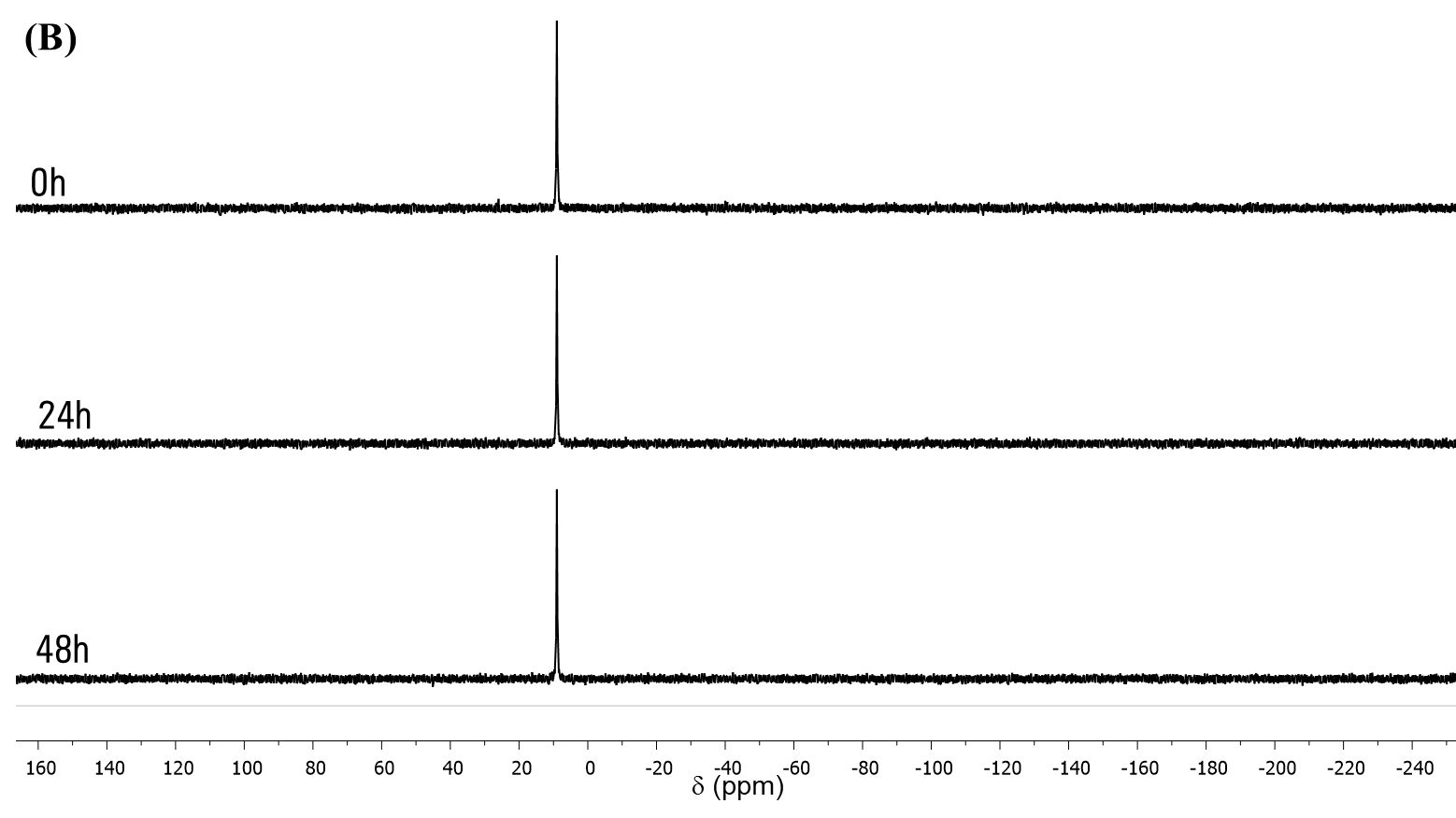 |
| 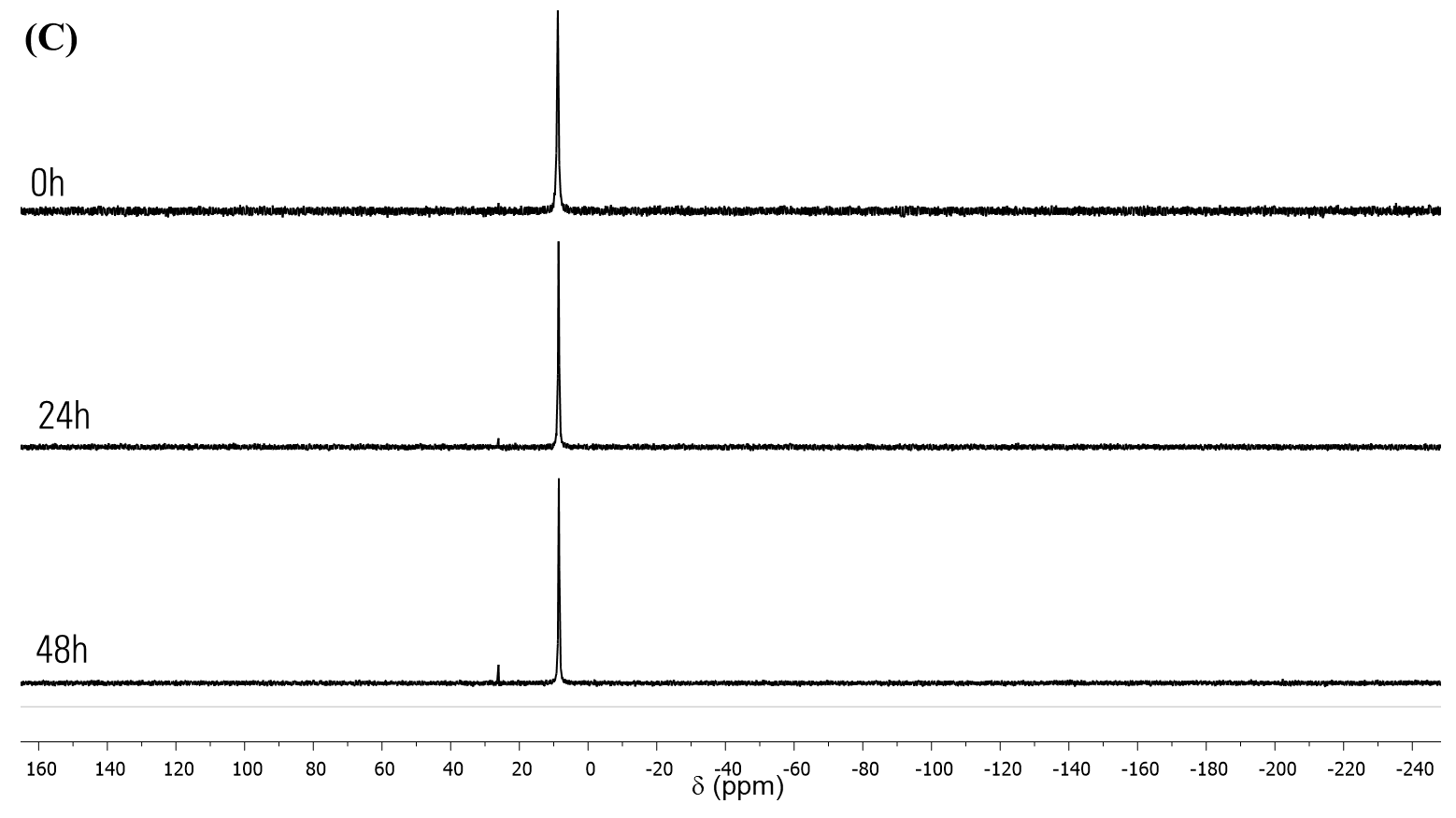 |

**Fig. S15.** ^31^P NMR spectra of complexes (A) **2**, (B) **3** and (C) **4** recorded in DMSO (using a capillary containing D_2_O) over a period of 48 hours.

|  |
| --- |
|  |
|  |
|  |
|  |

**Fig. S16.** Concentration-response curve of tumor cells (MDA-MB-231, MCF7, A549 and A2780cis) and non-tumor cells (MCF-10A and MRC-5) after complexes treatment for 48 h.

Table S1. Crystal data and structure refinement for complexes **2** and **3**.

| **Complex** | **2** | **3** |
| --- | --- | --- |
| CCDC code | 2415261 | 2415260 |
| Empirical formula | C_46_H_35_AgO_3_P_2_ | C_51_H_43_AgO_3_P_2_ |
| Formula weight | 805.604 | 873.66 |
| Temperature/K | 298(2) | 298(2) |
| Crystal system | triclinic | monoclinic |
| Space group | P-1 | C2/c |
| a/Å | 10.9566(4) | 25.8486(12) |
| b/Å | 12.8659(3) | 17.8158(10) |
| c/Å | 13.4077(3) | 18.6068(12) |
| α/° | 84.485(2) | 90 |
| β/° | 88.437(2) | 96.070(5) |
| γ/° | 83.202(2) | 90 |
| Volume/Å^3^ | 1867.82(9) | 8520.6(8) |
| Z | 2 | 8 |
| ρ_calc_g/cm^3^ | 1.432 | 1.362 |
| μ/mm^‑1^ | 0.667 | 0.591 |
| F(000) | 823.1 | 3600.0 |
| Crystal size/mm^3^ | 0.65 × 0.41 × 0.33 | 0.277 × 0.201 × 0.033 |
| Radiation | Mo Kα (λ = 0.71073) | Mo Kα (λ = 0.71073) |
| 2Θ range for data collection/° | 5.2 to 51.36 | 5.064 to 51.362 |
| Index ranges | -16 ≤ h ≤ 16, -20 ≤ k ≤ 20, -21 ≤ l ≤ 20 | -31 ≤ h ≤ 31, -21 ≤ k ≤ 21,  -22 ≤ l ≤ 22 |
| Reflections collected | 57901 | 83037 |
| Independent reflections | 7097 [R_int_ = 0.0251, R_sigma_ = 0.0242] | 8098 [R_int_ = 0.0884, R_sigma_ = 0.0381] |
| Data/restraints/parameters | 7097/0/545 | 8098/1/516 |
| Goodness-of-fit on F^2^ | 1.120 | 1.055 |
| Final R indexes [I>=2σ (I)] | R_1_ = 0.0217, wR_2_ = 0.0494 | R_1_ = 0.0492, wR_2_ = 0.1202 |
| Final R indexes [all data] | R_1_ = 0.0267, wR_2_ = 0.0558 | R_1_ = 0.0750, wR_2_ = 0.1424 |
| Largest diff. peak/hole / e Å^-3^ | 0.51/-0.22 | 1.14/-1.21 |

Table S2. Main Bond Angles for Complex **2** and **3**.

| Bond Angles (deg) | Complex 2 | Complex 3 |
| --- | --- | --- |
| P2–Ag1–P1 | 126.90(4) | 130.85(3) |
| P1–Ag1–O1 | 99.33(5) | 96.76(10) |
| P2–Ag1–O1 | 100.59(5) | 108.05(9) |
| P1–Ag1–O2 | 112.17(5) | 109.58(8) |
| P2–Ag1–O2 | 120.91(5) | 118.92(8) |
| O2–Ag1–O1 | 68.37(5) | 67.58(10) |

Table S3. Bond Lengths for complexes **2**.

| **Atom** | **Atom** | **Length/Å** | **Atom** | **Atom** | **Length/Å** |
| --- | --- | --- | --- | --- | --- |
| Ag1 | P1 | 2.4458(12) | C121 | C122 | 1.383(3) |
| Ag1 | P2 | 2.4365(12) | C121 | C126 | 1.384(3) |
| Ag1 | O1 | 2.5066(14) | C122 | C123 | 1.387(3) |
| Ag1 | O2 | 2.3036(13) | C123 | C124 | 1.364(4) |
| P1 | C111 | 1.816(2) | C124 | C125 | 1.366(4) |
| P1 | C121 | 1.826(2) | C125 | C126 | 1.375(3) |
| P1 | C131 | 1.816(2) | C131 | C132 | 1.380(3) |
| P2 | C211 | 1.818(2) | C131 | C136 | 1.390(3) |
| P2 | C221 | 1.823(2) | C132 | C133 | 1.381(3) |
| P2 | C231 | 1.822(2) | C133 | C134 | 1.362(4) |
| O1 | C1 | 1.217(2) | C134 | C135 | 1.361(4) |
| O2 | C2 | 1.266(2) | C135 | C136 | 1.383(3) |
| O4 | C4 | 1.236(3) | C211 | C212 | 1.385(3) |
| C1 | C2 | 1.512(3) | C211 | C216 | 1.383(3) |
| C1 | C10 | 1.470(2) | C212 | C213 | 1.377(3) |
| C2 | C3 | 1.374(3) | C213 | C214 | 1.368(3) |
| C3 | C4 | 1.412(3) | C214 | C215 | 1.364(3) |
| C4 | C5 | 1.487(3) | C215 | C216 | 1.381(3) |
| C5 | C6 | 1.382(3) | C221 | C222 | 1.388(3) |
| C5 | C10 | 1.392(3) | C221 | C226 | 1.381(3) |
| C6 | C7 | 1.371(4) | C222 | C223 | 1.378(3) |
| C7 | C8 | 1.365(4) | C223 | C224 | 1.376(3) |
| C8 | C9 | 1.384(3) | C224 | C225 | 1.368(3) |
| C9 | C10 | 1.380(3) | C225 | C226 | 1.389(3) |
| C111 | C112 | 1.387(3) | C231 | C232 | 1.380(3) |
| C111 | C116 | 1.388(3) | C231 | C236 | 1.387(3) |
| C112 | C113 | 1.382(3) | C232 | C233 | 1.382(3) |
| C113 | C114 | 1.368(3) | C233 | C234 | 1.369(3) |
| C114 | C115 | 1.372(3) | C234 | C235 | 1.363(3) |
| C115 | C116 | 1.374(3) | C235 | C236 | 1.378(3) |

Table S4. Bond Lengths for complexes **3**.

| **Atom** | **Atom** | **Length/Å** | **Atom** | **Atom** | **Length/Å** |
| --- | --- | --- | --- | --- | --- |
| Ag1 | P1 | 2.43001(10) | C114 | C115 | 1.369(9) |
| Ag1 | P2 | 2.4238(11) | C115 | C116 | 1.382(7) |
| Ag1 | O1 | 2.521(3) | C121 | C122 | 1.394(6) |
| Ag1 | O2 | 2.297(3) | C121 | C126 | 1.363(6) |
| P1 | C111 | 1.838(4) | C122 | C123 | 1.378(7) |
| P1 | C121 | 1.826(4) | C123 | C124 | 1.351(7) |
| P1 | C131 | 1.836(4) | C124 | C125 | 1.355(7) |
| P2 | C211 | 1.814(4) | C125 | C126 | 1.384(7) |
| P2 | C221 | 1.828(4) | C131 | C132 | 1.378(7) |
| P2 | C231 | 1.814(4) | C131 | C136 | 1.366(6) |
| O1 | C1 | 1.218(5) | C132 | C133 | 1.367(7) |
| O2 | C2 | 1.274(5) | C133 | C134 | 1.356(8) |
| O4 | C4 | 1.239(6) | C134 | C135 | 1.346(9) |
| C1 | C2 | 1.503(6) | C135 | C136 | 1.397(7) |
| C1 | C10 | 1.473(6) | C211 | C212 | 1.378(6) |
| C2 | C3 | 1.390(6) | C211 | C216 | 1.380(6) |
| C3 | C4 | 1.418(7) | C212 | C213 | 1.387(7) |
| C3 | C11 | 1.503(7) | C213 | C214 | 1.357(8) |
| C4 | C5 | 1.495(8) | C214 | C215 | 1.370(9) |
| C5 | C6 | 1.394(7) | C215 | C216 | 1.386(7) |
| C5 | C10 | 1.379(7) | C221 | C222 | 1.384(6) |
| C6 | C7 | 1.356(11) | C221 | C226 | 1.384(7) |
| C7 | C8 | 1.355(12) | C222 | C223 | 1.386(8) |
| C8 | C9 | 1.383(8) | C223 | C224 | 1.347(10) |
| C9 | C10 | 1.381(7) | C224 | C225 | 1.360(10) |
| C11 | C12 | 1.493(7) | C225 | C226 | 1.380(8) |
| C12 | C13 | 1.297(7) | C231 | C232 | 1.384(6) |
| C13 | C14 | 1.497(8) | C231 | C236 | 1.376(7) |
| C13 | C15 | 1.501(8) | C232 | C233 | 1.386(7) |
| C111 | C112 | 1.382(6) | C233 | C234 | 1.360(9) |
| C111 | C116 | 1.389(6) | C234 | C235 | 1.357(9) |
| C112 | C113 | 1.385(7) | C235 | C236 | 1.380(7) |
| C113 | C114 | 1.365(9) |  |  |  |
