# Supplementary material for: Ag(I) complexes with natural products and triphenylphosphine: activity against tumor cell lines and mycobacterium tuberculosis
Source: J Biol Inorg Chem. 2026 Apr 28;31(3):211–21. doi: 10.1007/s00775-026-02144-1 (PMC13287109; doi:10.1007/s00775-026-02144-1)

checkCIF/PLATON report

Structure factors have been supplied for datablock(s) AgLap

THIS REPORT IS FOR GUIDANCE ONLY. IF USED AS PART OF A REVIEW PROCEDURE FOR PUBLICATION, IT SHOULD NOT REPLACE THE EXPERTISE OF AN EXPERIENCED CRYSTALLOGRAPHIC REFEREE.

No syntax errors found. CIF dictionary Interpreting this report

Datablock: AgLap

Bond precision: C-C = 0.0081 A Wavelength=0.71073

Cell:	a=25.8486(12)	b=17.8158(10)	c=18.6068(12)
	alpha=90	beta=96.070(5)	gamma=90
Temperature:	298 K		

	Calculated	Reported
Volume	8520.6(8)	8520.6(8)
Space group	C 2/c	C 1 2/c 1
Hall group	-C 2yc	-C 2yc
Moiety formula	C51 H43 Ag O3 P2	C51 H43 Ag O3 P2
Sum formula	C51 H43 Ag O3 P2	C51 H43 Ag O3 P2
Mr	873.66	873.66
Dx,g cm-3	1.362	1.362
Z	8	8
Mu (mm-1)	0.591	0.591
F000	3600.0	3600.0
F000'	3595.70	
h,k,lmax	31,21,22	31,21,22
Nref	8104	8098
Tmin,Tmax	0.867,0.981	0.656,1.000
Tmin'	0.849	

Correction method= # Reported T Limits: Tmin=0.656 Tmax=1.000
AbsCorr = MULTI-SCAN

Data completeness= 0.999 Theta(max)= 25.681

R(reflections)= 0.0492(5918) wR2(reflections)= 0.1424(8098)

S = 1.055 Npar= 516

The following ALERTS were generated. Each ALERT has the format
test-name_ALERT_alert-type_alert-level.
Click on the hyperlinks for more details of the test.

Alert level C

```
PLAT241_ALERT_2_C High   'MainMol' Ueq as Compared to Neighbors of         O1 Check
PLAT241_ALERT_2_C High   'MainMol' Ueq as Compared to Neighbors of         C7 Check
PLAT241_ALERT_2_C High   'MainMol' Ueq as Compared to Neighbors of       C125 Check
PLAT242_ALERT_2_C Low    'MainMol' Ueq as Compared to Neighbors of        C13 Check
PLAT331_ALERT_2_C Small Aver Phenyl C-C Dist C131     --C136     .       1.37 Ang.
PLAT342_ALERT_3_C Low Bond Precision on  C-C Bonds ...............    0.00806 Ang.
PLAT905_ALERT_3_C Negative K value in the Analysis of Variance ...     -1.384 Report
PLAT910_ALERT_3_C Missing # of FCF Reflection(s) Below Theta(Min).          6 Note
                0  2  0,   1  1  0,   2  0  0,  -1  1  1,   1  1  1,   0  0  2,
```

Alert level G

```
PLAT002_ALERT_2_G Number of Distance or Angle Restraints on AtSite          2 Note
PLAT083_ALERT_2_G SHELXL Second Parameter in WGHT  Unusually Large      20.68 Why ?
PLAT172_ALERT_4_G The CIF-Embedded .res File Contains DFIX Records          1 Report
PLAT232_ALERT_2_G Hirshfeld Test Diff (M-X)  Ag1      --P1       .        9.7 s.u.
PLAT333_ALERT_2_G Large Aver C6-Ring C-C Dist C1       -C10      .       1.45 Ang.
PLAT860_ALERT_3_G Number of Least-Squares Restraints .............          1 Note
PLAT969_ALERT_5_G The 'Henn et al.' R-Factor-gap value ..........      3.543 Note
              Predicted wR2: Based on SigI**2  4.02 or SHELX Weight 13.50
PLAT978_ALERT_2_G Number C-C Bonds with Positive Residual Density.          1 Info
```

0 **ALERT level A** = Most likely a serious problem - resolve or explain
0 **ALERT level B** = A potentially serious problem, consider carefully
8 **ALERT level C** = Check. Ensure it is not caused by an omission or oversight
8 **ALERT level G** = General information/check it is not something unexpected

0 ALERT type 1 CIF construction/syntax error, inconsistent or missing data
10 ALERT type 2 Indicator that the structure model may be wrong or deficient
4 ALERT type 3 Indicator that the structure quality may be low
1 ALERT type 4 Improvement, methodology, query or suggestion
1 ALERT type 5 Informative message, check

It is advisable to attempt to resolve as many as possible of the alerts in all categories. Often the minor alerts point to easily fixed oversights, errors and omissions in your CIF or refinement strategy, so attention to these fine details can be worthwhile. In order to resolve some of the more serious problems it may be necessary to carry out additional measurements or structure refinements. However, the purpose of your study may justify the reported deviations and the more serious of these should normally be commented upon in the discussion or experimental section of a paper or in the "special_details" fields of the CIF. checkCIF was carefully designed to identify outliers and unusual parameters, but every test has its limitations and alerts that are not important in a particular case may appear. Conversely, the absence of alerts does not guarantee there are no aspects of the results needing attention. It is up to the individual to critically assess their own results and, if necessary, seek expert advice.

Publication of your CIF in IUCr journals

A basic structural check has been run on your CIF. These basic checks will be run on all CIFs submitted for publication in IUCr journals (*Acta Crystallographica*, *Journal of Applied Crystallography*, *Journal of Synchrotron Radiation*); however, if you intend to submit to *Acta Crystallographica Section C* or *E* or *IUCrData*, you should make sure that full publication checks are run on the final version of your CIF prior to submission.

Publication of your CIF in other journals

Please refer to the *Notes for Authors* of the relevant journal for any special instructions relating to CIF submission.

PLATON version of 02/02/2025; check.def file version of 02/02/2025

Datablock AgLap - ellipsoid plot

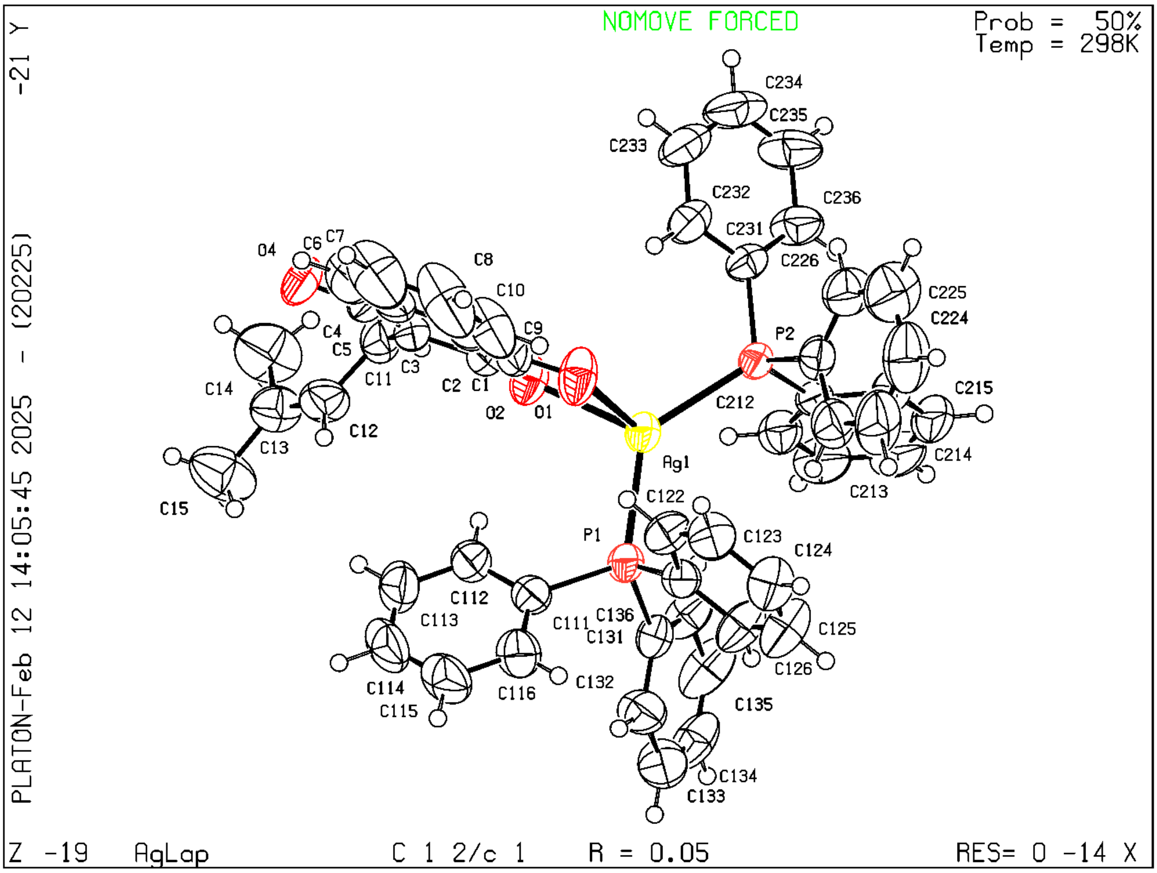

Supplement: Supplementary file 2 — Supplementary Material 2 [file 775_2026_2144_MOESM2_ESM.pdf]
